# Seasonality, control, and risk factors for *Gasterophilus intestinalis* egg intensity in horses from Romania under field conditions

**DOI:** 10.1007/s00436-025-08540-x

**Published:** 2025-08-06

**Authors:** Ș.O. Rabei, D. Pivariu, A. I. Cocian, D. Vaccaro, P. Costache-Bobescu, A. D. Mihalca

**Affiliations:** 1 Department of Parasitology and Parasitic Diseases, Faculty of Veterinary Medicine, University of Agricultural Sciences and Veterinary Medicine of Cluj-Napoca, 400372 Cluj-Napoca, Romania; 2 Department of Toxicology, Faculty of Veterinary Medicine, University of Agricultural Sciences and Veterinary Medicine of Cluj-Napoca, 400372 Cluj-Napoca, Romania; 3 Ceva Sante Animale Romania, Strada Chindiei Nr. 5, Sector 4, 040185 Bucharest, Romania; 4 Parasitology Consultancy Group, 407056 Corușu, Romania

**Keywords:** *Gasterophilus intestinalis*, Eggs, Repellent, Seasonality, Color, Husbandry

## Abstract

This study aimed to assess the efficacy of pyrethroid-based insecticides against *Gasterophilus* spp. egg-laying activity and to establish the seasonality patterns in the specific climatic area of Transylvania, Romania. To fulfil the aims of the study, a total of 40 horses were treated every 4 weeks and inspected every 2 weeks between 11 May and 6 November (Pilo et al. Parasitol Res 114:1693–1702, 2024). Through this period, eggs were found between 24 June and 2 November. The eggs were found on 34 out of 40 horses (prevalence = 85%, CI 95% = 73.43–96.56%). Overall, a total of 50,029 *Gasterophilus* spp. eggs were counted during this study, and all were morphologically identified as *G. intestinalis*. Statistical analysis revealed no significant difference in prevalence and intensity between treated and control horses (*p* > 0.05). The number of laid eggs varied significantly across body regions (*p* < 0.05). The risk factors associated with increased egg-laying activity were significantly (*p* < 0.05) associated with dark-color coats, outside husbandry regime, and the month of exposure. No significant differences in egg numbers and prevalence were found across gender or age groups (*p* > 0.05). The applied statistical tests were as follows: the non-parametric Mann–Whitney *U* test, the Friedman test, Wilcoxon test corrected by the Bonferroni method, the Kruskal–Wallis test, and the Bonferroni correction for multiple comparisons. These results highlight gasterophilosis as an important parasite in horses. In conclusion, we recommend that, in order maintain a low prevalence of *Gasterophilus* spp., treatment should include the use of larvicidal drugs and the mechanical removal of the eggs.

**Supplementary Information:**

The online version contains supplementary material available at 10.1007/s00436-025-08540-x.

## Background

*Gasterophilus* spp. are widespread parasites that cause digestive myiasis in several species of equids (Zumpt 1965; Colwell et al. 2006). Occasionally, *Gasterophilus* spp. were also found in non-equid hosts such as lions (Ndossi et al. 2024), wolves (Uakhit et al. 2025), cattle (Cope and Catts 1991), but also humans (Anderson 2006; Sun et al. 2025). Since the description of the genus, there has been a constant change in the number of *Gasterophilus* species recognized as valid (Li et al. 2019). At the same time, data on distribution, prevalence, or intensity are continuously changing due to new studies but also environmental or human-related factors (Otranto et al. 2005).

The life cycle of *Gasterophilus* spp. lasts almost 12 months, while the adult lifespan can reach 26 days. The pupal period lasts around 34 days, and eggs become ready to hatch within 10 days after deposition (Colwell et al. 2006). Both periods were shown to be highly dependent on weather conditions, restricted to minimum temperature thresholds and humidity values. The development rate, and therefore the intensity of larvae, is increasingly concomitant with temperature values (Sukhapesna et al. 1975; Zhang et al. 2021, 2024; Huang et al. 2022). Consequently, in response to climate changes, the epidemiology of *Gasterophilus* spp. may differ considerably from one generation to another. In addition, anthropogenic factors may either prevent the spread of the infection through controlled activities such as antiparasitic treatment and removal of the *Gasterophilus* eggs or on the contrary contribute to the spreading of *Gasterophilus* spp. through improper use of antiparasitic drugs or relocation of horses between different epidemiological areas (Otranto et al. 2005).

The distribution of *Gasterophilus* spp. varies significantly between areas due to intrinsic and extrinsic factors. In Europe, *G. intestinalis* and *G. nasalis* are the most common species, while *G. haemorrhoidalis*, *G. inermis*, *G. pecorum*, *G. meridionalis*, *G. nigricornis*, and *G. flavipes* are less spread and usually less abundant (Otranto et al. 2005; Rodrigues Felix et al. 2007; Rehbein et al. 2013; Rabei et al. 2024).

The pathology varies mainly according to the sites and intensity of infestation. The signs associated with the presence of *Gasterophilus* spp. larvae in the gastrointestinal tract range from asymptomatic infection to severe disorders associated with malabsorption of nutrients and lesions in the secretory structures of the gastrointestinal mucosa induced by the mechanical activity of larvae (Principato 1988; Cogley and Cogley 1999; Sequeira et al. 2001; Otranto et al. 2005; Pilo et al. 2015; Liu et al. 2016). *G. inermis* has been described as a cause of dermatitis (Carbonell et al. 2023). In addition to the above detrimental effects, adults have also been proven to be a stress factor for horses on pastures (Cogley and Cogley 1999). Hence, the appropriate approach to prevent the negative impact upon horses should address all stages of the *Gasterophilus* spp. life cycle.

To date, only a few clinical studies evaluated the efficacy of antiparasitic drugs against *Gasterophilus* spp. Macrocyclic lactone–based endectocides, such as ivermectin (AbdElKader et al. 2021) and moxidectin (Scholl et al. 1998), have been proven highly efficient against larvae of *G. intestinalis* and *G. nasalis*. Additionally, natural substances like curcumin have shown a high efficacy (Attia et al. 2022) against larvae. Some pyrethroid-based products are labelled to be efficient against adult *Gasterophilus* spp. (Vet-Kem® Flea—piperonyl butoxide, N-Octyl bicycloheptene dicarboximide, s-metrophene; Flygon Gold®—piperonyl butoxide, permethrin, pyrethrin—none registered in EU), but scientific data is largely missing on the efficacy of insecticides against adults, which is evaluated in the current study.

It is assumed that *G. intestinalis* females are seeking their hosts through their olfactive and vision senses (Cope and Catts 1991; Cogley and Cogley 1999; Zhang et al. 2022). The records presented in literature suggest that *G. intestinalis* females are more attracted to the horses that are found in open areas that the ones kept in stalls or darker spaces (Cogley and Cogley 1999).

Considering the limited data on the effects of pyrethroids against *Gasterophilus* spp. adults and the relatively scarce field data of *Gasterophilus* spp. in Eastern Europe, the aim of this study was to evaluate the efficacy of a commercial product containing transmethrin and tetramethrin against the infestation with *G. intestinalis* eggs under natural field conditions using a randomized clinical study. At the same time, we aimed to establish the seasonality patterns of *Gasterophilus* spp. in the specific climatic area of Transylvania, Romania.

## Material and methods

### Study design

Based on our previous epidemiological data on the seasonality of larvae (Rabei et al. 2024), the field study was initiated on 11 May 2024. The egg-laying activity was evaluated by performing egg counts on all horses every 14 ± 2 days, until two consecutive negative results were noted (which occurred in the first half of November 2024). For the assessment of the efficacy of transmethrin and tetramethrin under field conditions, all horses were randomly assigned (block randomization) to one of the two different groups: first group (treated group) and second group (control group). The study had a single-blind design. The horses included in the first group were treated (according to the label) with a commercial product containing transmethrin and tetramethrin every 28 days until the end of the study. For each control date, we recorded the health status of horses and the precise number of eggs on horses’ coats, counted using photographs (single observer). All *Gasterophilus* eggs were removed from the horse coat after each treatment in the treated group, and at the corresponding follow-up visit in the control group. From each positive horse, at each sampling time, 1–5 eggs were randomly collected and stored in 70% ethanol eggs for further identification.

### Study area and horses

The field study was implemented within Cluj County boundaries in an area of approximately 100 km^2^. The weather in Romania is shaped by both temperate and continental climates, with four seasons (winter, spring, summer, and autumn) and with various climatic influences: Oceanic, Mediterranean, Baltic, and Pontic. The region of Transylvania classified as humid continental climate (Dfb class) (Peel et al. 2007) has an average daily temperature of 16 °C, with mean negative values only in January. Mean daily temperatures over 10 °C are recorded in the interval April–November with the peak mean of 25.1 °C in July. The relative humidity ranges from 74 to 87% with the lowest values in June and July and the highest value in December (www.meteoromania.ro). The inclusion dates were 11^th^, 12^th^, and 25^th^ of May 2024. A total of 60 horses, aged between 2 months and 25 years, were included in the study, but only 40 completed the study (20 horses dropped out of the study because of various reasons, such as owner compliance, and movement of animals to different areas or to different owners). Included horses were kept outdoor or indoor-outdoor (details included in supplementary file 1).

### Inclusion, exclusion, and removal criteria

The owners were informed about the requirements of the study, and after they signed an informed consent, their horses were clinically inspected. Only horses that were clinically healthy, that have not been treated with other insecticides within the last 60 days, and were at least 8 weeks old, were included in the study. Horses were randomly assigned to groups, except for lactating mares, which were included in the control group.

Exclusion criteria included horses under the age of 8 weeks and horses with evident clinical conditions or which were treated with any insecticide within the last 60 days.

After inclusion, horses that changed their owner, had their health status or behavior altered because of other causes or were treated with other ectoparasiticides, were removed from the study.

Morphological species identification of *Gasterophilus* eggs.

The morphological characteristics of each egg were observed under an Olympus SZX16 stereo-zoom microscope. Microphotographs were taken with an Olympus SC180 camera, and dedicated software. The eggs were identified according to morphological keys (Cogley 1991; Li et al. 2019).

### Statistical analysis

The number of eggs was recorded at each 14 (± 2) days for both groups, and the specific body regions were recorded. For statistical analysis, the horses were grouped into three age groups (< 3 years, 3–10 years, > 10 years) and two color categories (1 – light colored (i.e., white, gray), 2 – dark colored (i.e., bay, brown, roan, black)). Egg counts were recorded separately for five body regions as follows: head and neck, thorax, abdomen, foreleg, and hindleg. Additionally, the foreleg was also split into three distinct counting zones: 1 – carpus and metacarpus, 2 – fetlock and pastern, 3 – rest of the foreleg (see Fig. 6).

The non-parametric Mann–Whitney *U* test was performed to assess the preventive efficacy of the pyrethroid against the presence of *Gasterophilus* spp. eggs. The Friedman test was used to compare the mean intensity between both body regions and control dates. Post hoc comparisons were performed using the Wilcoxon test corrected by the Bonferroni method. Other statistical tests were performed to assess the differences of prevalence, mean intensity, and mean abundance across date, age, gender, color, and husbandry practices. The statistical significance between prevalence and host data and date was assessed by performing chi-square (χ2) test, while the relationship between egg mean intensity and mean abundance across host data was analyzed using the Mann–Whitney and Kruskal–Wallis tests. Following the Mann–Whitney *U* test results, post hoc multiple comparisons were performed using Bonferroni correction. The statistical tests mentioned above were performed using IBM S.P.S.S. 20.0 software.

## Results

**Fig. 1:**
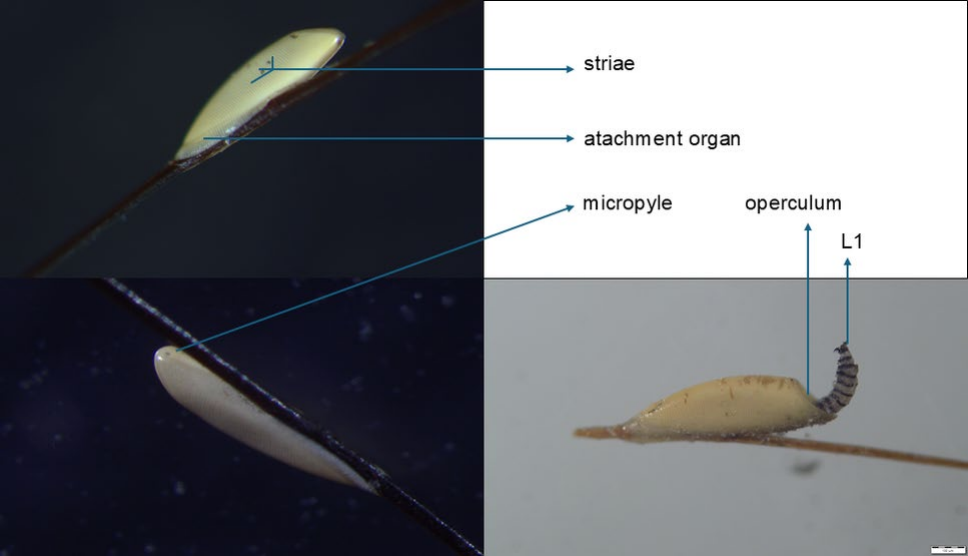
*G. intestinalis* egg morphology

The treated group consisted of 22 horses, while the control group included 18 horses. Out of the 40 horses (aged between 8 weeks and 23 years, 23 females, 17 males) which completed the study, 34 had at least once *G. intestinalis* eggs (prevalence = 85%, CI 95% = 73.43–96.56%) during the evaluation period. Overall, a total of 50,029 *Gasterophilus* spp. eggs were counted during this study. All randomly collected eggs were identified as *G. intestinalis* (Fig. 1). *G. intestinalis* eggs were found on horses from 24 June until 2 November.

**Fig. 2:**
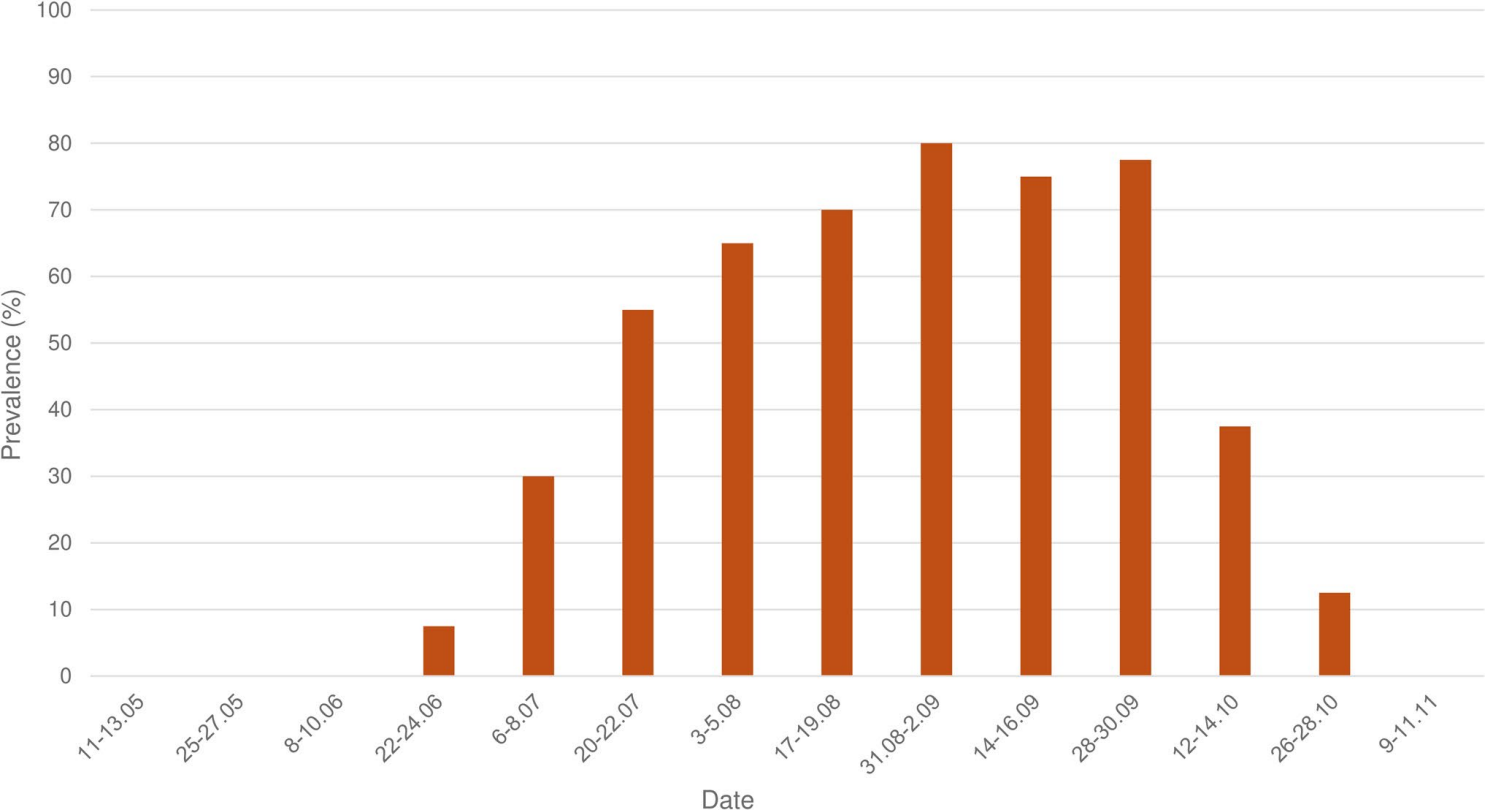
*G. intestinalis* eggs prevalence across control dates

There was no statistical difference between the prevalence in the treated group (86.36%, CI 95% = 70.79–100) and the control group (83.33%, CI 95% = 64.26–100). The chi-square (χ2) test showed significant differences between prevalence and month (χ2 = 107.083, df = 9, *p* = 0.000), reaching its peak in September (Fig. 2). Significant differences were also present in relation to husbandry practices (χ2 = 21.190, df = 1, *p* = 0.000), with a higher prevalence for horses which were kept outdoor. For the other categories of variables (host sex, age, color), there were no significant differences.

**Fig. 3:**
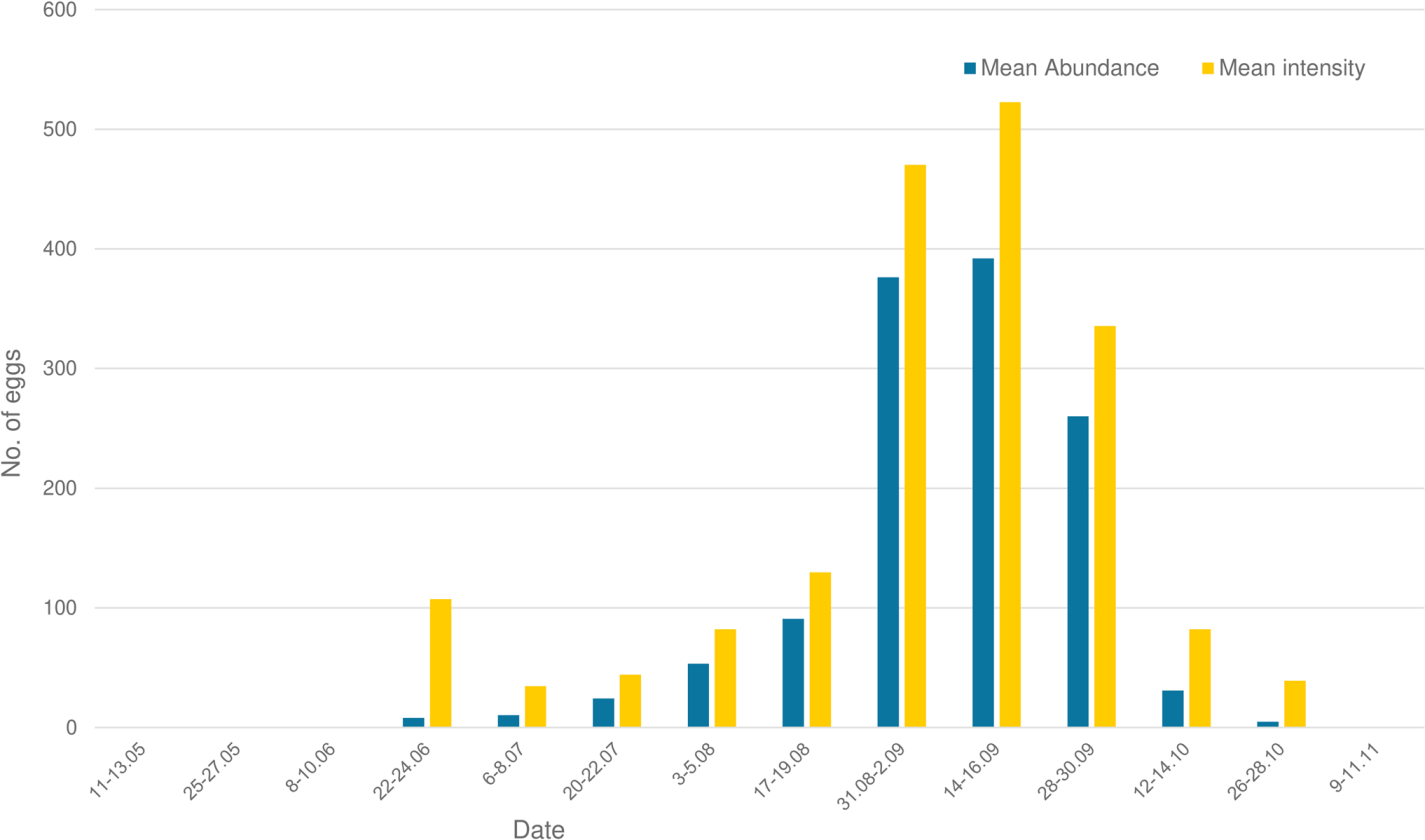
*G. intestinalis* eggs mean intensity and mean abundance across control dates

**Fig. 4:**
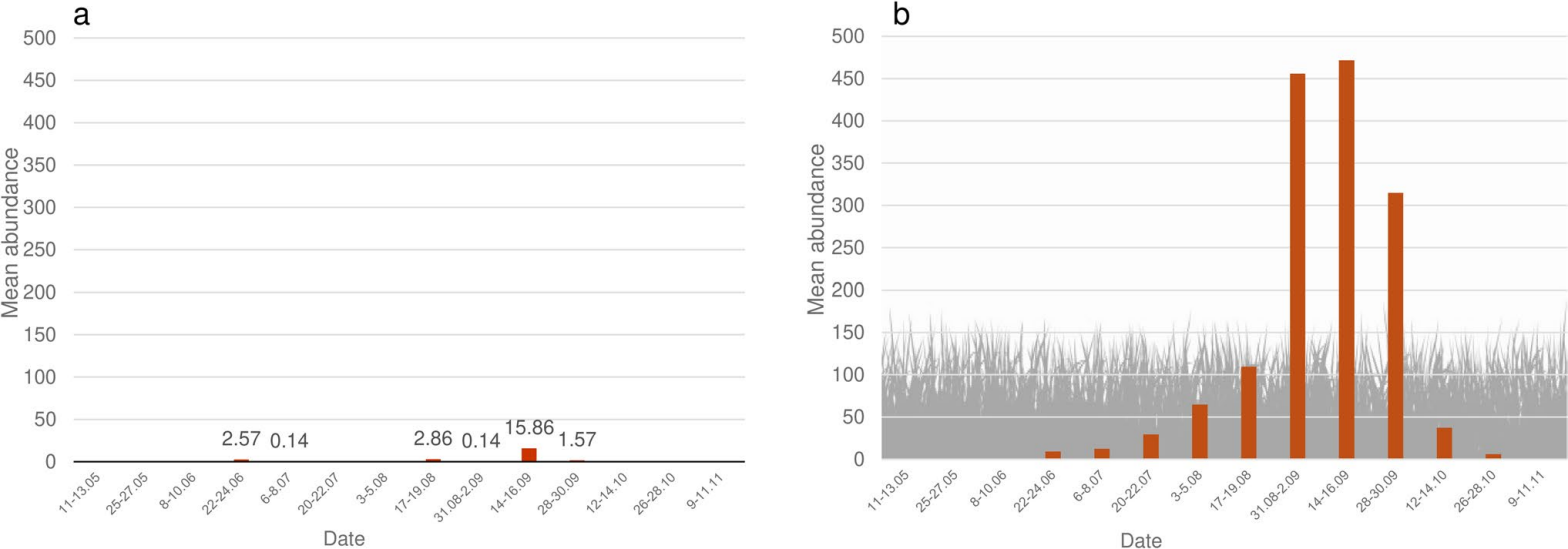
*G. intestinalis* eggs mean abundance across husbandry groups. **a** Indoor-outdoor. **b** Outdoor only

**Fig. 5:**
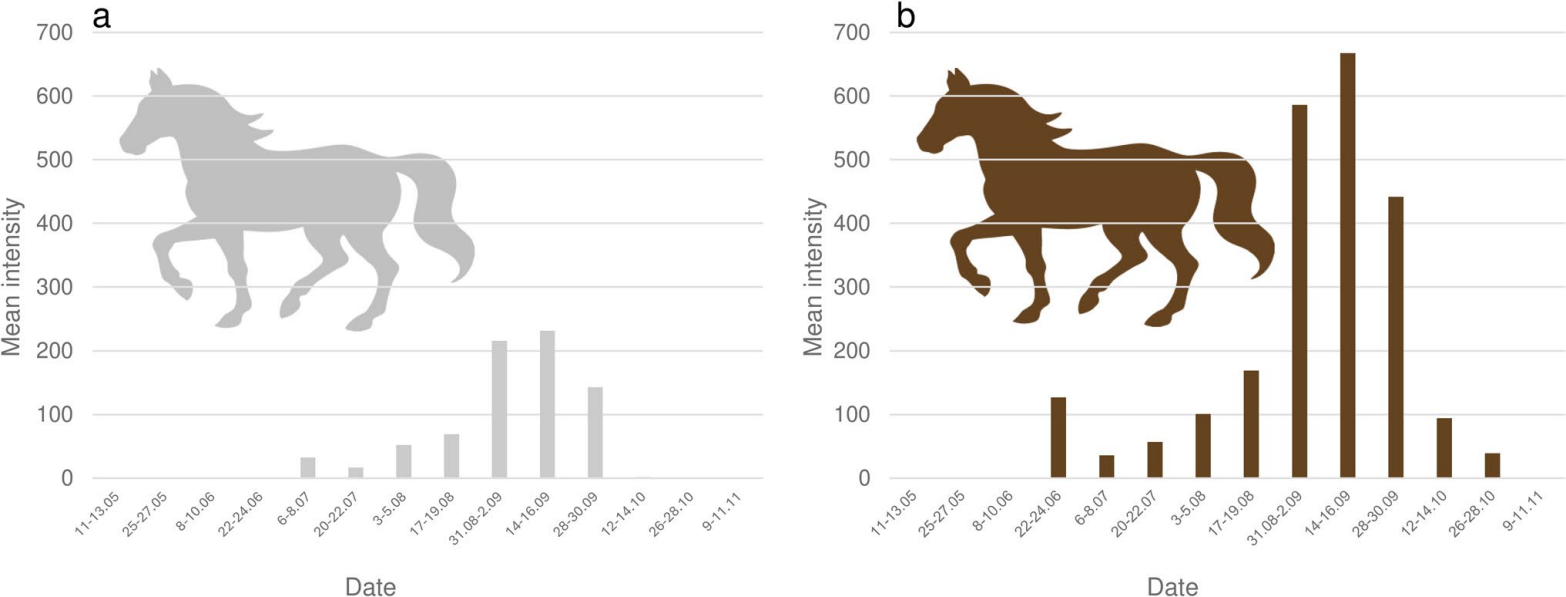
*G. intestinalis* eggs mean intensity across color groups. **a** Light colored. **b** Dark colored

The Mann–Whitney *U* test revealed no statistically significant differences across the values of mean intensity and mean abundance of eggs between the treated and control groups. However, the Mann–Whitney *U* test revealed significant differences for the mean abundance for month (*p* < 0.05) (Fig. 3) and husbandry practices (*p* < 0.05). The same test showed significant differences also between the values of mean intensity across month (*p* < 0.05) (Fig. 3), husbandry practices (*p* < 0.05), and color (*p* < 0.05). *G. intestinalis* females were more likely to lay their eggs on horses that were kept outdoors (Fig. 4) while the number of the eggs laid on lighter coat was significantly lower than in dark-colored horses (Fig. 5).

**Fig. 6:**
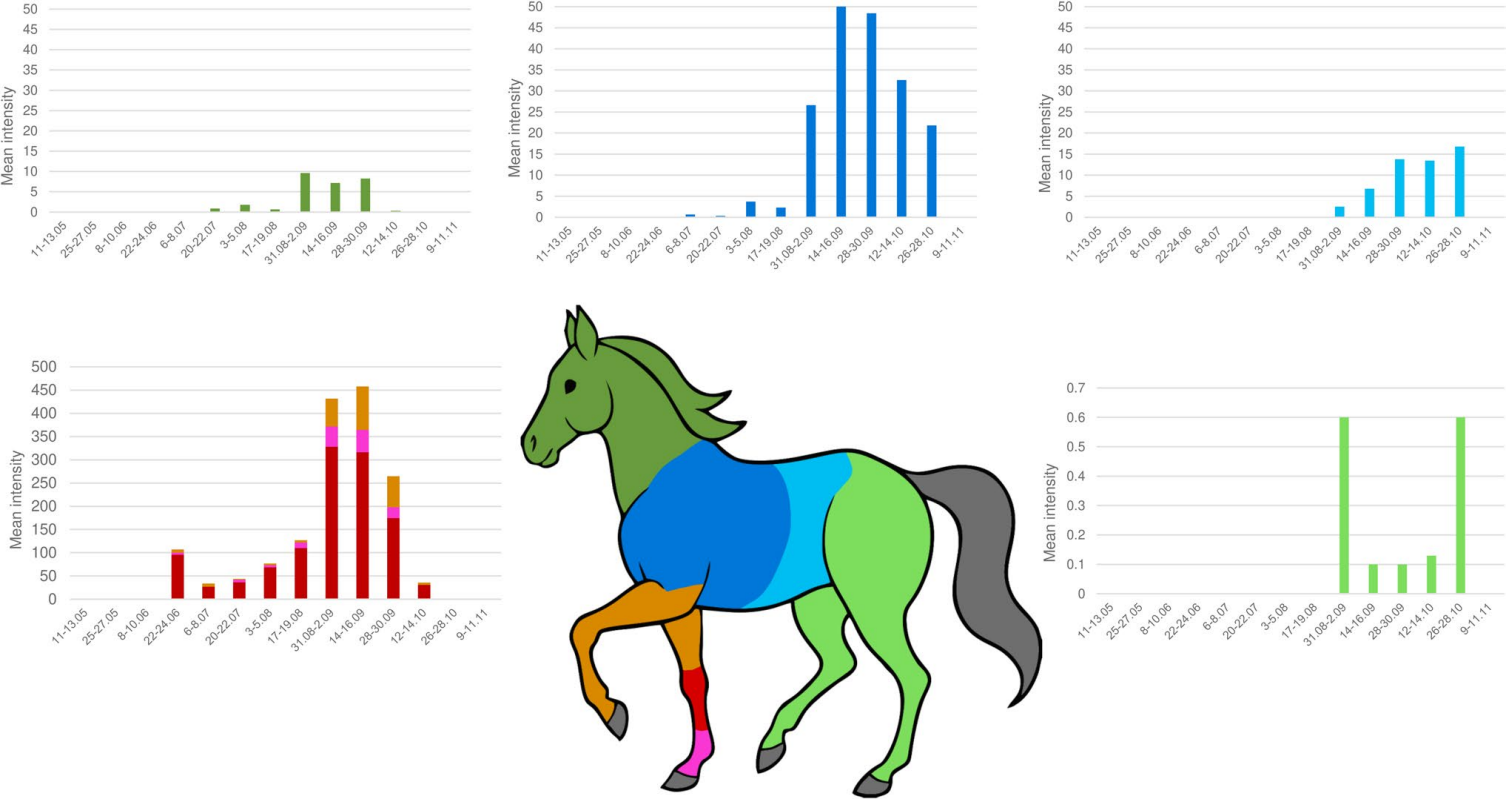
*G. intestinalis* eggs mean intensity across anatomical regions (all X axes show the date of examination)

Analysis of egg deposition patterns using the Friedman test indicated a significant difference in *G. intestinalis* female choice for the body region for egg-laying (χ2 (4) = 102.700, *p* < 0.001). Moreover, the same test revealed a significant difference between the regions of the foreleg with the medial carpus and metacarpus area is the election site for egg-laying activity (χ2 (2) = 44.176, *p* < 0.001). Furthermore, the results for the post hoc comparison using the Wilcoxon test corrected by the Bonferroni method showed differences between the foreleg and all the other body regions (*p* < 0.05) and between the hindleg and all the other body regions (*p* < 0.05), except head and neck area. The mean intensity values by anatomical region and month are displayed in Fig. 6.

The detailed results of pairwise comparison with Bonferroni correction for the months and the anatomical regions as well as the other statistical tests are displayed in Supplementary material 2.

## Discussion

This is the first study to evaluate the risk factors associated with the application of repellents on the prevalence and intensity of eggs of *Gasterophilus* spp. However, the application of a commercial combination of tetramethrin and transmethrin did not decrease the risk of infestation of horses with eggs of *G. intestinalis*. *Gasterophilus* spp. females are most probably using visual and olfactory stimuli to detect and select the host for egg-laying (Cope and Catts 1991; Cogley and Cogley 2000; Zhang et al. 2022). Other parasitic flies with horses as preferred hosts (e.g., tabanids) are attracted by odors spread through breath, urine, skin, or feces or by distinct coat patterns (Horváth et al. 2010; Blahó et al. 2013).

Our study is the first to quantitatively evaluate the seasonal dynamics and other risk factors for the prevalence and intensity of *Gasterophilus* spp. eggs on horses in areas with continental climate of Europe. Although expected, our study also demonstrated for the first time that horses kept outdoors are at a significantly higher risk of getting infected with *G. intestinalis*.

The high prevalence of eggs found in this study is in accordance with our previous results on the seasonality of larvae in horses examined in the slaughterhouses from Romania (Rabei et al. 2024). Although no quantitative evaluations are available, data from Switzerland (Brocard and Pfister 1991) indicate a similar pattern, with the presence of eggs between July and November. Cope and Catts (1991) found eggs from June to September in Delaware, USA, Doby (1987) between July and October in France, and Sánchez-Andrade et al. (2010) between June and September in Spain. Other studies from Europe indicated the peak season of eggs in summer-autumn in Italy, based on extrapolation of necropsy data (Otranto et al. 2005). The peak prevalence of *Gasterophilus* spp. eggs in northern England and Wales was in September (Edwards 1982).

Our study showed that the predilection site for egg-laying activity of *G. intestinalis* is the foreleg (87% of the eggs), supporting other data (Brocard and Pfister 1991; Cope and Catts 1991; Cogley and Cogley 1999; Pilo et al. 2015). This may be explained by the fact that the eggs deposited on the foreleg are easier to ingest than those elsewhere. However, at the last positive check on 2 November, eggs were present only in thoracic and abdominal regions.

Contrary with the results obtained by Pilo et al. 2015, our study found no significant difference between the amount off eggs laid on the coat of males and females or across age classes. Additionally, we observed significant differences between color groups and husbandry types. The preference of darker coat color was also presented by Pilo et al. (2009) and by Cogley and Cogley (2000). Still, the influence of color was not demonstrated in other studies (Pandey et al. 1980; Brocard and Pfister 1991).

Even though *Gasterophilus* spp. larvae are sensitive to larvicide drugs (Scholl et al. 1998; AbdElKader et al. 2021; Attia et al. 2022), the behavioral characteristics of adult females of *Gasterophilus* spp. and consequently the egg-laying activity are dependent on various risk factors which are host, husbandry, or environmentally related. All these contribute to the maintenance of a high prevalence and high intensity in certain seasons.

## Conclusion

A high prevalence of *G. intestinalis* eggs was found in our study. The statistical comparison between the control group and the treated group showed no significant differences and therefore revealed that pyrethroids are not effective against the infestation with *G. intestinalis* eggs. The peak of egg-laying activity in both prevalence and mean intensity was in September (Fig. 2, Fig. 3). Statistically significant results showed that dark-color horses and outdoor kept horses are more predisposed to the infestation. Currently, there is no drug available for the prevention of egg-laying activity by female *Gasterophilus* in Europe. Despite the limited data on the assessment of gasterophilosis-related health issues in horses, we consider that infestation represents a welfare concern in our country and elsewhere. Therefore, the appropriate control of gasterophilosis should include the mechanical removal of eggs, particularly in horses with outdoor access and in high-risk months (i.e., September).

## Supplementary Information

Below is the link to the electronic supplementary material.

Supplementary file1 (XLSX 86 KB)

Supplementary file2 (DOCX 261 KB)

## Data Availability

No datasets were generated or analysed during the current study.
