# Supplementary material for: Seasonality, control, and risk factors for Gasterophilus intestinalis egg intensity in horses from Romania under field conditions
Source: Parasitol Res. 2025 Aug 6;124(8):87. doi: 10.1007/s00436-025-08540-x (PMC12328499; doi:10.1007/s00436-025-08540-x)
Supplement: Supplementary file 2 — Supplementary file2 (DOCX 261 KB) [file 436_2025_8540_MOESM2_ESM.docx]

Contents

[1. Treatment Group 2](#_Toc194549309)

[1.1. Prevalance 2](#_Toc194549310)

[1.2. Mean Abundance 2](#_Toc194549311)

[1.3. Mean Intensity 2](#_Toc194549312)

[2. Date 2](#_Toc194549313)

[2.1. Prevalence – significant 2](#_Toc194549314)

[2.2. Mean Abundance - significant 2](#_Toc194549315)

[2.3. Mean intensity – significant 2](#_Toc194549316)

[3. Gender 2](#_Toc194549317)

[3.1. Prevalence - significant 2](#_Toc194549318)

[3.2. Mean Abundance - significant 2](#_Toc194549319)

[3.3. Mean intensity 2](#_Toc194549320)

[4. Age Group 2](#_Toc194549321)

[4.1. Prevalence 2](#_Toc194549322)

[4.2. Mean Abundance 2](#_Toc194549323)

[4.3. Mean intensity 2](#_Toc194549324)

[5. Colour Group 2](#_Toc194549325)

[5.1. Prevalence 2](#_Toc194549326)

[5.2. Mean Abundance 2](#_Toc194549327)

[5.3. Mean intensity - significant 2](#_Toc194549328)

[6. Husbandry 2](#_Toc194549329)

[6.1. Prevalence – significant 2](#_Toc194549330)

[6.2. Mean Abundance -significant 2](#_Toc194549331)

[6.3. Mean intensity - significant 2](#_Toc194549332)

[7. Anatomical Regions 2](#_Toc194549333)

[7.1. Mean intensity – anatomical regions – significant 2](#_Toc194549334)

[7.2. Mean intensity – foreleg regions – significant 2](#_Toc194549335)

# Treatment Group

## Prevalance

| Positive * Treat. Gr. Crosstabulation | | | | | |
| --- | --- | --- | --- | --- | --- |
| Count | | | | | |
|  | | |  | |  |
| Date | | | Treat. Gr. | | Total |
|  |  |  | Control | Treated |  |
| 28-30.09 | Positive | 0 | 4 | 5 | 9 |
|  |  | 1 | 14 | 17 | 31 |
|  | Total | | 18 | 22 | 40 |
| 12-14.10 | Positive | 0 | 12 | 13 | 25 |
|  |  | 1 | 6 | 9 | 15 |
|  | Total | | 18 | 22 | 40 |
| 26-28.10 | Positive | 0 | 15 | 20 | 35 |
|  |  | 1 | 3 | 2 | 5 |
|  | Total | | 18 | 22 | 40 |
| 22-24.06 | Positive | 0 | 18 | 19 | 37 |
|  |  | 1 | 0 | 3 | 3 |
|  | Total | | 18 | 22 | 40 |
| 6-8.07 | Positive | 0 | 12 | 16 | 28 |
|  |  | 1 | 6 | 6 | 12 |
|  | Total | | 18 | 22 | 40 |
| 20-22.07 | Positive | 0 | 11 | 7 | 18 |
|  |  | 1 | 7 | 15 | 22 |
|  | Total | | 18 | 22 | 40 |
| 3-5.08 | Positive | 0 | 5 | 9 | 14 |
|  |  | 1 | 13 | 13 | 26 |
|  | Total | | 18 | 22 | 40 |
| 17-19.08 | Positive | 0 | 7 | 5 | 12 |
|  |  | 1 | 11 | 17 | 28 |
|  | Total | | 18 | 22 | 40 |
| 31.08-2.09 | Positive | 0 | 3 | 5 | 8 |
|  |  | 1 | 15 | 17 | 32 |
|  | Total | | 18 | 22 | 40 |
| 14-16.09 | Positive | 0 | 6 | 4 | 10 |
|  |  | 1 | 12 | 18 | 30 |
|  | Total | | 18 | 22 | 40 |
| Total | Positive | 0 | 3 | 3 | 6 |
|  |  | 1 | 15 | 19 | 34 |
|  | Total | | 18 | 22 | 40 |

| **Chi-Square Tests** | | | | | | |
| --- | --- | --- | --- | --- | --- | --- |
| Date | | Value | df | Asymptotic Significance (2-sided) | Exact Sig. (2-sided) | Exact Sig. (1-sided) |
| 28-30.09 | Pearson Chi-Square | .001^a^ | 1 | .970 |  |  |
|  | Continuity Correction^b^ | .000 | 1 | 1.000 |  |  |
|  | Likelihood Ratio | .001 | 1 | .970 |  |  |
|  | Fisher's Exact Test |  |  |  | 1.000 | .636 |
|  | N of Valid Cases | 40 |  |  |  |  |
| 12-14.10 | Pearson Chi-Square | .242^c^ | 1 | .622 |  |  |
|  | Continuity Correction^b^ | .027 | 1 | .870 |  |  |
|  | Likelihood Ratio | .243 | 1 | .622 |  |  |
|  | Fisher's Exact Test |  |  |  | .747 | .436 |
|  | N of Valid Cases | 40 |  |  |  |  |
| 26-28.10 | Pearson Chi-Square | .519^d^ | 1 | .471 |  |  |
|  | Continuity Correction^b^ | .058 | 1 | .810 |  |  |
|  | Likelihood Ratio | .517 | 1 | .472 |  |  |
|  | Fisher's Exact Test |  |  |  | .642 | .402 |
|  | N of Valid Cases | 40 |  |  |  |  |
| 22-24.06 | Pearson Chi-Square | 2.654^e^ | 1 | .103 |  |  |
|  | Continuity Correction^b^ | 1.052 | 1 | .305 |  |  |
|  | Likelihood Ratio | 3.785 | 1 | .052 |  |  |
|  | Fisher's Exact Test |  |  |  | .238 | .156 |
|  | N of Valid Cases | 40 |  |  |  |  |
| 6-8.07 | Pearson Chi-Square | .173^f^ | 1 | .677 |  |  |
|  | Continuity Correction^b^ | .005 | 1 | .945 |  |  |
|  | Likelihood Ratio | .173 | 1 | .678 |  |  |
|  | Fisher's Exact Test |  |  |  | .738 | .471 |
|  | N of Valid Cases | 40 |  |  |  |  |
| 20-22.07 | Pearson Chi-Square | 3.432^g^ | 1 | .064 |  |  |
|  | Continuity Correction^b^ | 2.351 | 1 | .125 |  |  |
|  | Likelihood Ratio | 3.473 | 1 | .062 |  |  |
|  | Fisher's Exact Test |  |  |  | .110 | .062 |
|  | N of Valid Cases | 40 |  |  |  |  |
| 3-5.08 | Pearson Chi-Square | .750^h^ | 1 | .386 |  |  |
|  | Continuity Correction^b^ | .284 | 1 | .594 |  |  |
|  | Likelihood Ratio | .758 | 1 | .384 |  |  |
|  | Fisher's Exact Test |  |  |  | .510 | .298 |
|  | N of Valid Cases | 40 |  |  |  |  |
| 17-19.08 | Pearson Chi-Square | 1.231^f^ | 1 | .267 |  |  |
|  | Continuity Correction^b^ | .582 | 1 | .446 |  |  |
|  | Likelihood Ratio | 1.230 | 1 | .267 |  |  |
|  | Fisher's Exact Test |  |  |  | .315 | .223 |
|  | N of Valid Cases | 40 |  |  |  |  |
| 31.08-2.09 | Pearson Chi-Square | .227^i^ | 1 | .634 |  |  |
|  | Continuity Correction^b^ | .006 | 1 | .937 |  |  |
|  | Likelihood Ratio | .230 | 1 | .632 |  |  |
|  | Fisher's Exact Test |  |  |  | .709 | .472 |
|  | N of Valid Cases | 40 |  |  |  |  |
| 14-16.09 | Pearson Chi-Square | 1.212^j^ | 1 | .271 |  |  |
|  | Continuity Correction^b^ | .539 | 1 | .463 |  |  |
|  | Likelihood Ratio | 1.210 | 1 | .271 |  |  |
|  | Fisher's Exact Test |  |  |  | .300 | .231 |
|  | N of Valid Cases | 40 |  |  |  |  |
| Total | Pearson Chi-Square | .071^k^ | 1 | .789 |  |  |
|  | Continuity Correction^b^ | .000 | 1 | 1.000 |  |  |
|  | Likelihood Ratio | .071 | 1 | .790 |  |  |
|  | Fisher's Exact Test |  |  |  | 1.000 | .565 |
|  | N of Valid Cases | 40 |  |  |  |  |
| a. 2 cells (50.0%) have expected count less than 5. The minimum expected count is 4.05. | | | | | | |
| b. Computed only for a 2x2 table | | | | | | |
| c. 0 cells (0.0%) have expected count less than 5. The minimum expected count is 6.75. | | | | | | |
| d. 2 cells (50.0%) have expected count less than 5. The minimum expected count is 2.25. | | | | | | |
| e. 2 cells (50.0%) have expected count less than 5. The minimum expected count is 1.35. | | | | | | |
| f. 0 cells (0.0%) have expected count less than 5. The minimum expected count is 5.40. | | | | | | |
| g. 0 cells (0.0%) have expected count less than 5. The minimum expected count is 8.10. | | | | | | |
| h. 0 cells (0.0%) have expected count less than 5. The minimum expected count is 6.30. | | | | | | |
| i. 2 cells (50.0%) have expected count less than 5. The minimum expected count is 3.60. | | | | | | |
| j. 1 cells (25.0%) have expected count less than 5. The minimum expected count is 4.50. | | | | | | |
| k. 2 cells (50.0%) have expected count less than 5. The minimum expected count is 2.70. | | | | | | |

| **Chi-Square Tests** | | | | | |
| --- | --- | --- | --- | --- | --- |
|  | Value | df | Asymptotic Significance (2-sided) | Exact Sig. (2-sided) | Exact Sig. (1-sided) |
| Pearson Chi-Square | .962^a^ | 1 | .327 |  |  |
| Continuity Correction^b^ | .782 | 1 | .376 |  |  |
| Likelihood Ratio | .962 | 1 | .327 |  |  |
| Fisher's Exact Test |  |  |  | .338 | .188 |
| N of Valid Cases | 440 |  |  |  |  |
| a. 0 cells (0.0%) have expected count less than 5. The minimum expected count is 90.90. | | | | | |
| b. Computed only for a 2x2 table | | | | | |

## Mean Abundance

| **Independent-Samples Mann-Whitney U Test Summary** | | |
| --- | --- | --- |
| 28-30.09 | Total N | 40 |
|  | Mann-Whitney U | 230.000 |
|  | Wilcoxon W | 483.000 |
|  | Test Statistic | 230.000 |
|  | Standard Error | 36.576 |
|  | Standardized Test Statistic | .875 |
|  | Asymptotic Sig.(2-sided test) | .382 |
|  | Exact Sig.(2-sided test) | .396 |
| 12-14.10 | Total N | 40 |
|  | Mann-Whitney U | 209.500 |
|  | Wilcoxon W | 462.500 |
|  | Test Statistic | 209.500 |
|  | Standard Error | 31.982 |
|  | Standardized Test Statistic | .360 |
|  | Asymptotic Sig.(2-sided test) | .719 |
|  | Exact Sig.(2-sided test) | .757 |
| 26-28.10 | Total N | 40 |
|  | Mann-Whitney U | 185.000 |
|  | Wilcoxon W | 438.000 |
|  | Test Statistic | 185.000 |
|  | Standard Error | 21.137 |
|  | Standardized Test Statistic | -.615 |
|  | Asymptotic Sig.(2-sided test) | .539 |
|  | Exact Sig.(2-sided test) | .737 |
| 22-24.06 | Total N | 40 |
|  | Mann-Whitney U | 225.000 |
|  | Wilcoxon W | 478.000 |
|  | Test Statistic | 225.000 |
|  | Standard Error | 16.801 |
|  | Standardized Test Statistic | 1.607 |
|  | Asymptotic Sig.(2-sided test) | .108 |
|  | Exact Sig.(2-sided test) | .476 |
| 6-8.07 | Total N | 40 |
|  | Mann-Whitney U | 192.500 |
|  | Wilcoxon W | 445.500 |
|  | Test Statistic | 192.500 |
|  | Standard Error | 29.818 |
|  | Standardized Test Statistic | -.184 |
|  | Asymptotic Sig.(2-sided test) | .854 |
|  | Exact Sig.(2-sided test) | .882 |
| 20-22.07 | Total N | 40 |
|  | Mann-Whitney U | 266.500 |
|  | Wilcoxon W | 519.500 |
|  | Test Statistic | 266.500 |
|  | Standard Error | 35.070 |
|  | Standardized Test Statistic | 1.953 |
|  | Asymptotic Sig.(2-sided test) | .051 |
|  | Exact Sig.(2-sided test) | .062 |
| 3-5.08 | Total N | 40 |
|  | Mann-Whitney U | 158.000 |
|  | Wilcoxon W | 411.000 |
|  | Test Statistic | 158.000 |
|  | Standard Error | 35.988 |
|  | Standardized Test Statistic | -1.111 |
|  | Asymptotic Sig.(2-sided test) | .266 |
|  | Exact Sig.(2-sided test) | .286 |
| 17-19.08 | Total N | 40 |
|  | Mann-Whitney U | 254.500 |
|  | Wilcoxon W | 507.500 |
|  | Test Statistic | 254.500 |
|  | Standard Error | 36.285 |
|  | Standardized Test Statistic | 1.557 |
|  | Asymptotic Sig.(2-sided test) | .119 |
|  | Exact Sig.(2-sided test) | .125 |
| 31.08-2.09 | Total N | 40 |
|  | Mann-Whitney U | 223.500 |
|  | Wilcoxon W | 476.500 |
|  | Test Statistic | 223.500 |
|  | Standard Error | 36.638 |
|  | Standardized Test Statistic | .696 |
|  | Asymptotic Sig.(2-sided test) | .486 |
|  | Exact Sig.(2-sided test) | .492 |
| 14-16.09 | Total N | 40 |
|  | Mann-Whitney U | 234.000 |
|  | Wilcoxon W | 487.000 |
|  | Test Statistic | 234.000 |
|  | Standard Error | 36.496 |
|  | Standardized Test Statistic | .986 |
|  | Asymptotic Sig.(2-sided test) | .324 |
|  | Exact Sig.(2-sided test) | .338 |
| Total | Total N | 40 |
|  | Mann-Whitney U | 234.500 |
|  | Wilcoxon W | 487.500 |
|  | Test Statistic | 234.500 |
|  | Standard Error | 36.723 |
|  | Standardized Test Statistic | .994 |
|  | Asymptotic Sig.(2-sided test) | .320 |
|  | Exact Sig.(2-sided test) | .325 |

## Mean Intensity

| **Hypothesis Test Summary** | | | | | | |
| --- | --- | --- | --- | --- | --- | --- |
| Date | Positive | | Null Hypothesis | Test | Sig.^a,b^ | Decision |
| 28-30.09 | 0 | 1 | The distribution of Total is the same across categories of Treat. Gr.. | Independent-Samples Mann-Whitney U Test | 1.000^c^ | Retain the null hypothesis. |
|  | 1 | 1 | The distribution of Total is the same across categories of Treat. Gr.. | Independent-Samples Mann-Whitney U Test | .200^c^ | Retain the null hypothesis. |
| 12-14.10 | 0 | 1 | The distribution of Total is the same across categories of Treat. Gr.. | Independent-Samples Mann-Whitney U Test | 1.000^c^ | Retain the null hypothesis. |
|  | 1 | 1 | The distribution of Total is the same across categories of Treat. Gr.. | Independent-Samples Mann-Whitney U Test | .689^c^ | Retain the null hypothesis. |
| 26-28.10 | 0 | 1 | The distribution of Total is the same across categories of Treat. Gr.. | Independent-Samples Mann-Whitney U Test | 1.000^c^ | Retain the null hypothesis. |
|  | 1 | 1 | The distribution of Total is the same across categories of Treat. Gr.. | Independent-Samples Mann-Whitney U Test | .400^c^ | Retain the null hypothesis. |
| 22-24.06 | 0 | 1 | The distribution of Total is the same across categories of Treat. Gr.. | Independent-Samples Mann-Whitney U Test | 1.000^c^ | Retain the null hypothesis. |
| 6-8.07 | 0 | 1 | The distribution of Total is the same across categories of Treat. Gr.. | Independent-Samples Mann-Whitney U Test | 1.000^c^ | Retain the null hypothesis. |
|  | 1 | 1 | The distribution of Total is the same across categories of Treat. Gr.. | Independent-Samples Mann-Whitney U Test | .310^c^ | Retain the null hypothesis. |
| 20-22.07 | 0 | 1 | The distribution of Total is the same across categories of Treat. Gr.. | Independent-Samples Mann-Whitney U Test | 1.000^c^ | Retain the null hypothesis. |
|  | 1 | 1 | The distribution of Total is the same across categories of Treat. Gr.. | Independent-Samples Mann-Whitney U Test | .490^c^ | Retain the null hypothesis. |
| 3-5.08 | 0 | 1 | The distribution of Total is the same across categories of Treat. Gr.. | Independent-Samples Mann-Whitney U Test | 1.000^c^ | Retain the null hypothesis. |
|  | 1 | 1 | The distribution of Total is the same across categories of Treat. Gr.. | Independent-Samples Mann-Whitney U Test | .479^c^ | Retain the null hypothesis. |
| 17-19.08 | 0 | 1 | The distribution of Total is the same across categories of Treat. Gr.. | Independent-Samples Mann-Whitney U Test | 1.000^c^ | Retain the null hypothesis. |
|  | 1 | 1 | The distribution of Total is the same across categories of Treat. Gr.. | Independent-Samples Mann-Whitney U Test | .264^c^ | Retain the null hypothesis. |
| 31.08-2.09 | 0 | 1 | The distribution of Total is the same across categories of Treat. Gr.. | Independent-Samples Mann-Whitney U Test | 1.000^c^ | Retain the null hypothesis. |
|  | 1 | 1 | The distribution of Total is the same across categories of Treat. Gr.. | Independent-Samples Mann-Whitney U Test | .165^c^ | Retain the null hypothesis. |
| 14-16.09 | 0 | 1 | The distribution of Total is the same across categories of Treat. Gr.. | Independent-Samples Mann-Whitney U Test | 1.000^c^ | Retain the null hypothesis. |
|  | 1 | 1 | The distribution of Total is the same across categories of Treat. Gr.. | Independent-Samples Mann-Whitney U Test | .819^c^ | Retain the null hypothesis. |
| Total | 0 | 1 | The distribution of Total is the same across categories of Treat. Gr.. | Independent-Samples Mann-Whitney U Test | 1.000^c^ | Retain the null hypothesis. |
|  | 1 | 1 | The distribution of Total is the same across categories of Treat. Gr.. | Independent-Samples Mann-Whitney U Test | .302^c^ | Retain the null hypothesis. |
| a. The significance level is .050. | | | | | | |
| b. Asymptotic significance is displayed. | | | | | | |
| c. Exact significance is displayed for this test. | | | | | | |

**Independent-Samples Mann-Whitney U Test**

**Total across Treat. Gr.**

| **Independent-Samples Mann-Whitney U Test Summary** | | | |
| --- | --- | --- | --- |
| 28-30.09 | 0 | Total N | 9 |
|  |  | Mann-Whitney U | 10.000 |
|  |  | Wilcoxon W | 25.000 |
|  |  | Test Statistic | 10.000 |
|  |  | Standard Error | .000 |
|  |  | Standardized Test Statistic | .000 |
|  |  | Asymptotic Sig.(2-sided test) | 1.000 |
|  |  | Exact Sig.(2-sided test) | 1.000 |
|  | 1 | Total N | 31 |
|  |  | Mann-Whitney U | 152.000 |
|  |  | Wilcoxon W | 305.000 |
|  |  | Test Statistic | 152.000 |
|  |  | Standard Error | 25.193 |
|  |  | Standardized Test Statistic | 1.310 |
|  |  | Asymptotic Sig.(2-sided test) | .190 |
|  |  | Exact Sig.(2-sided test) | .200 |
| 12-14.10 | 0 | Total N | 25 |
|  |  | Mann-Whitney U | 78.000 |
|  |  | Wilcoxon W | 169.000 |
|  |  | Test Statistic | 78.000 |
|  |  | Standard Error | .000 |
|  |  | Standardized Test Statistic | .000 |
|  |  | Asymptotic Sig.(2-sided test) | 1.000 |
|  |  | Exact Sig.(2-sided test) | 1.000 |
|  | 1 | Total N | 15 |
|  |  | Mann-Whitney U | 23.500 |
|  |  | Wilcoxon W | 68.500 |
|  |  | Test Statistic | 23.500 |
|  |  | Standard Error | 8.478 |
|  |  | Standardized Test Statistic | -.413 |
|  |  | Asymptotic Sig.(2-sided test) | .680 |
|  |  | Exact Sig.(2-sided test) | .689 |
| 26-28.10 | 0 | Total N | 35 |
|  |  | Mann-Whitney U | 150.000 |
|  |  | Wilcoxon W | 360.000 |
|  |  | Test Statistic | 150.000 |
|  |  | Standard Error | .000 |
|  |  | Standardized Test Statistic | .000 |
|  |  | Asymptotic Sig.(2-sided test) | 1.000 |
|  |  | Exact Sig.(2-sided test) | 1.000 |
|  | 1 | Total N | 5 |
|  |  | Mann-Whitney U | 5.000 |
|  |  | Wilcoxon W | 8.000 |
|  |  | Test Statistic | 5.000 |
|  |  | Standard Error | 1.732 |
|  |  | Standardized Test Statistic | 1.155 |
|  |  | Asymptotic Sig.(2-sided test) | .248 |
|  |  | Exact Sig.(2-sided test) | .400 |
| 22-24.06 | 0 | Total N | 37 |
|  |  | Mann-Whitney U | 171.000 |
|  |  | Wilcoxon W | 361.000 |
|  |  | Test Statistic | 171.000 |
|  |  | Standard Error | .000 |
|  |  | Standardized Test Statistic | .000 |
|  |  | Asymptotic Sig.(2-sided test) | 1.000 |
|  |  | Exact Sig.(2-sided test) | 1.000 |
| 6-8.07 | 0 | Total N | 28 |
|  |  | Mann-Whitney U | 96.000 |
|  |  | Wilcoxon W | 232.000 |
|  |  | Test Statistic | 96.000 |
|  |  | Standard Error | .000 |
|  |  | Standardized Test Statistic | .000 |
|  |  | Asymptotic Sig.(2-sided test) | 1.000 |
|  |  | Exact Sig.(2-sided test) | 1.000 |
|  | 1 | Total N | 12 |
|  |  | Mann-Whitney U | 24.500 |
|  |  | Wilcoxon W | 45.500 |
|  |  | Test Statistic | 24.500 |
|  |  | Standard Error | 6.234 |
|  |  | Standardized Test Statistic | 1.043 |
|  |  | Asymptotic Sig.(2-sided test) | .297 |
|  |  | Exact Sig.(2-sided test) | .310 |
| 20-22.07 | 0 | Total N | 18 |
|  |  | Mann-Whitney U | 38.500 |
|  |  | Wilcoxon W | 66.500 |
|  |  | Test Statistic | 38.500 |
|  |  | Standard Error | .000 |
|  |  | Standardized Test Statistic | .000 |
|  |  | Asymptotic Sig.(2-sided test) | 1.000 |
|  |  | Exact Sig.(2-sided test) | 1.000 |
|  | 1 | Total N | 22 |
|  |  | Mann-Whitney U | 63.000 |
|  |  | Wilcoxon W | 183.000 |
|  |  | Test Statistic | 63.000 |
|  |  | Standard Error | 14.182 |
|  |  | Standardized Test Statistic | .740 |
|  |  | Asymptotic Sig.(2-sided test) | .459 |
|  |  | Exact Sig.(2-sided test) | .490 |
| 3-5.08 | 0 | Total N | 14 |
|  |  | Mann-Whitney U | 22.500 |
|  |  | Wilcoxon W | 67.500 |
|  |  | Test Statistic | 22.500 |
|  |  | Standard Error | .000 |
|  |  | Standardized Test Statistic | .000 |
|  |  | Asymptotic Sig.(2-sided test) | 1.000 |
|  |  | Exact Sig.(2-sided test) | 1.000 |
|  | 1 | Total N | 26 |
|  |  | Mann-Whitney U | 70.500 |
|  |  | Wilcoxon W | 161.500 |
|  |  | Test Statistic | 70.500 |
|  |  | Standard Error | 19.497 |
|  |  | Standardized Test Statistic | -.718 |
|  |  | Asymptotic Sig.(2-sided test) | .473 |
|  |  | Exact Sig.(2-sided test) | .479 |
| 17-19.08 | 0 | Total N | 12 |
|  |  | Mann-Whitney U | 17.500 |
|  |  | Wilcoxon W | 32.500 |
|  |  | Test Statistic | 17.500 |
|  |  | Standard Error | .000 |
|  |  | Standardized Test Statistic | .000 |
|  |  | Asymptotic Sig.(2-sided test) | 1.000 |
|  |  | Exact Sig.(2-sided test) | 1.000 |
|  | 1 | Total N | 28 |
|  |  | Mann-Whitney U | 118.000 |
|  |  | Wilcoxon W | 271.000 |
|  |  | Test Statistic | 118.000 |
|  |  | Standard Error | 21.255 |
|  |  | Standardized Test Statistic | 1.153 |
|  |  | Asymptotic Sig.(2-sided test) | .249 |
|  |  | Exact Sig.(2-sided test) | .264 |
| 31.08-2.09 | 0 | Total N | 8 |
|  |  | Mann-Whitney U | 7.500 |
|  |  | Wilcoxon W | 22.500 |
|  |  | Test Statistic | 7.500 |
|  |  | Standard Error | .000 |
|  |  | Standardized Test Statistic | .000 |
|  |  | Asymptotic Sig.(2-sided test) | 1.000 |
|  |  | Exact Sig.(2-sided test) | 1.000 |
|  | 1 | Total N | 32 |
|  |  | Mann-Whitney U | 165.000 |
|  |  | Wilcoxon W | 318.000 |
|  |  | Test Statistic | 165.000 |
|  |  | Standard Error | 26.481 |
|  |  | Standardized Test Statistic | 1.416 |
|  |  | Asymptotic Sig.(2-sided test) | .157 |
|  |  | Exact Sig.(2-sided test) | .165 |
| 14-16.09 | 0 | Total N | 10 |
|  |  | Mann-Whitney U | 12.000 |
|  |  | Wilcoxon W | 22.000 |
|  |  | Test Statistic | 12.000 |
|  |  | Standard Error | .000 |
|  |  | Standardized Test Statistic | .000 |
|  |  | Asymptotic Sig.(2-sided test) | 1.000 |
|  |  | Exact Sig.(2-sided test) | 1.000 |
|  | 1 | Total N | 30 |
|  |  | Mann-Whitney U | 114.000 |
|  |  | Wilcoxon W | 285.000 |
|  |  | Test Statistic | 114.000 |
|  |  | Standard Error | 23.619 |
|  |  | Standardized Test Statistic | .254 |
|  |  | Asymptotic Sig.(2-sided test) | .799 |
|  |  | Exact Sig.(2-sided test) | .819 |
| Total | 0 | Total N | 6 |
|  |  | Mann-Whitney U | 4.500 |
|  |  | Wilcoxon W | 10.500 |
|  |  | Test Statistic | 4.500 |
|  |  | Standard Error | .000 |
|  |  | Standardized Test Statistic | .000 |
|  |  | Asymptotic Sig.(2-sided test) | 1.000 |
|  |  | Exact Sig.(2-sided test) | 1.000 |
|  | 1 | Total N | 34 |
|  |  | Mann-Whitney U | 173.000 |
|  |  | Wilcoxon W | 363.000 |
|  |  | Test Statistic | 173.000 |
|  |  | Standard Error | 28.831 |
|  |  | Standardized Test Statistic | 1.058 |
|  |  | Asymptotic Sig.(2-sided test) | .290 |
|  |  | Exact Sig.(2-sided test) | .302 |

# Date

## Prevalence – significant

| **Date * Positive Crosstabulation** | | | | |
| --- | --- | --- | --- | --- |
| Count | | | | |
|  | | Positive | | Total |
|  |  | 0 | 1 |  |
| Date | 28-30.09 | 9 | 31 | 40 |
|  | 12-14.10 | 25 | 15 | 40 |
|  | 26-28.10 | 35 | 5 | 40 |
|  | 22-24.06 | 37 | 3 | 40 |
|  | 6-8.07 | 28 | 12 | 40 |
|  | 20-22.07 | 18 | 22 | 40 |
|  | 3-5.08 | 14 | 26 | 40 |
|  | 17-19.08 | 12 | 28 | 40 |
|  | 31.08-2.09 | 8 | 32 | 40 |
|  | 14-16.09 | 10 | 30 | 40 |
| Total | | 196 | 204 | 400 |

| **Chi-Square Tests** | | | |
| --- | --- | --- | --- |
|  | Value | df | Asymptotic Significance (2-sided) |
| Pearson Chi-Square | 107.083^a^ | 9 | .000 |
| Likelihood Ratio | 117.723 | 9 | .000 |
| N of Valid Cases | 400 |  |  |
| a. 0 cells (0.0%) have expected count less than 5. The minimum expected count is 19.60. | | | |

## Mean Abundance – significant

| **Hypothesis Test Summary** | | | | |
| --- | --- | --- | --- | --- |
|  | Null Hypothesis | Test | Sig.^a,b^ | Decision |
| 1 | The distribution of Total is the same across categories of Date. | Independent-Samples Kruskal-Wallis Test | .000 | Reject the null hypothesis. |
| a. The significance level is .050. | | | | |
| b. Asymptotic significance is displayed. | | | | |

| **Independent-Samples Kruskal-Wallis Test Summary** | |
| --- | --- |
| Total N | 400 |
| Test Statistic | 140.645^a^ |
| Degree Of Freedom | 9 |
| Asymptotic Sig.(2-sided test) | .000 |
| a. The test statistic is adjusted for ties. | |

| Pairwise Comparisons of Date | | | | | |
| --- | --- | --- | --- | --- | --- |
| Sample 1-Sample 2 | Test Statistic | Std. Error | Std. Test Statistic | Sig. | Adj. Sig.^a^ |
| 22-24.06-26-28.10 | -4.900 | 24.284 | -.202 | .840 | 1.000 |
| 22-24.06-6-8.07 | -27.412 | 24.284 | -1.129 | .259 | 1.000 |
| 22-24.06-12-14.10 | -49.650 | 24.284 | -2.045 | .041 | 1.000 |
| 22-24.06-20-22.07 | -68.150 | 24.284 | -2.806 | .005 | .225 |
| 22-24.06-3-5.08 | -103.600 | 24.284 | -4.266 | .000 | .001 |
| 22-24.06-17-19.08 | -121.487 | 24.284 | -5.003 | .000 | .000 |
| 22-24.06-28-30.09 | -161.737 | 24.284 | -6.660 | .000 | .000 |
| 22-24.06-14-16.09 | -172.325 | 24.284 | -7.096 | .000 | .000 |
| 22-24.06-31.08-2.09 | -173.862 | 24.284 | -7.160 | .000 | .000 |
| 26-28.10-6-8.07 | 22.513 | 24.284 | .927 | .354 | 1.000 |
| 26-28.10-12-14.10 | 44.750 | 24.284 | 1.843 | .065 | 1.000 |
| 26-28.10-20-22.07 | 63.250 | 24.284 | 2.605 | .009 | .414 |
| 26-28.10-3-5.08 | 98.700 | 24.284 | 4.064 | .000 | .002 |
| 26-28.10-17-19.08 | 116.588 | 24.284 | 4.801 | .000 | .000 |
| 26-28.10-28-30.09 | 156.838 | 24.284 | 6.459 | .000 | .000 |
| 26-28.10-14-16.09 | 167.425 | 24.284 | 6.895 | .000 | .000 |
| 26-28.10-31.08-2.09 | 168.963 | 24.284 | 6.958 | .000 | .000 |
| 6-8.07-12-14.10 | -22.237 | 24.284 | -.916 | .360 | 1.000 |
| 6-8.07-20-22.07 | -40.737 | 24.284 | -1.678 | .093 | 1.000 |
| 6-8.07-3-5.08 | -76.187 | 24.284 | -3.137 | .002 | .077 |
| 6-8.07-17-19.08 | -94.075 | 24.284 | -3.874 | .000 | .005 |
| 6-8.07-28-30.09 | -134.325 | 24.284 | -5.531 | .000 | .000 |
| 6-8.07-14-16.09 | -144.912 | 24.284 | -5.967 | .000 | .000 |
| 6-8.07-31.08-2.09 | -146.450 | 24.284 | -6.031 | .000 | .000 |
| 12-14.10-20-22.07 | 18.500 | 24.284 | .762 | .446 | 1.000 |
| 12-14.10-3-5.08 | 53.950 | 24.284 | 2.222 | .026 | 1.000 |
| 12-14.10-17-19.08 | 71.838 | 24.284 | 2.958 | .003 | .139 |
| 12-14.10-28-30.09 | 112.088 | 24.284 | 4.616 | .000 | .000 |
| 12-14.10-14-16.09 | 122.675 | 24.284 | 5.052 | .000 | .000 |
| 12-14.10-31.08-2.09 | 124.213 | 24.284 | 5.115 | .000 | .000 |
| 20-22.07-3-5.08 | -35.450 | 24.284 | -1.460 | .144 | 1.000 |
| 20-22.07-17-19.08 | -53.337 | 24.284 | -2.196 | .028 | 1.000 |
| 20-22.07-28-30.09 | -93.587 | 24.284 | -3.854 | .000 | .005 |
| 20-22.07-14-16.09 | -104.175 | 24.284 | -4.290 | .000 | .001 |
| 20-22.07-31.08-2.09 | -105.712 | 24.284 | -4.353 | .000 | .001 |
| 3-5.08-17-19.08 | -17.887 | 24.284 | -.737 | .461 | 1.000 |
| 3-5.08-28-30.09 | -58.137 | 24.284 | -2.394 | .017 | .750 |
| 3-5.08-14-16.09 | -68.725 | 24.284 | -2.830 | .005 | .209 |
| 3-5.08-31.08-2.09 | -70.262 | 24.284 | -2.893 | .004 | .171 |
| 17-19.08-28-30.09 | -40.250 | 24.284 | -1.657 | .097 | 1.000 |
| 17-19.08-14-16.09 | -50.837 | 24.284 | -2.093 | .036 | 1.000 |
| 17-19.08-31.08-2.09 | -52.375 | 24.284 | -2.157 | .031 | 1.000 |
| 28-30.09-14-16.09 | 10.588 | 24.284 | .436 | .663 | 1.000 |
| 28-30.09-31.08-2.09 | 12.125 | 24.284 | .499 | .618 | 1.000 |
| 14-16.09-31.08-2.09 | 1.538 | 24.284 | .063 | .950 | 1.000 |
| Each row tests the null hypothesis that the Sample 1 and Sample 2 distributions are the same.  Asymptotic significances (2-sided tests) are displayed. The significance level is .050. | | | | | |
| a. Significance values have been adjusted by the Bonferroni correction for multiple tests. | | | | | |

## Mean intensity – significant

| **Hypothesis Test Summary** | | | | | |
| --- | --- | --- | --- | --- | --- |
| Positive | | Null Hypothesis | Test | Sig.^a,b^ | Decision |
| 0 | 1 | The distribution of Total is the same across categories of Date. | Independent-Samples Kruskal-Wallis Test | 1.000 | Retain the null hypothesis. |
| 1 | 1 | The distribution of Total is the same across categories of Date. | Independent-Samples Kruskal-Wallis Test | .000 | Reject the null hypothesis. |
| a. The significance level is .050. | | | | | |
| b. Asymptotic significance is displayed. | | | | | |

| **Independent-Samples Kruskal-Wallis Test Summary** | | | | | | |  |  |  |
| --- | --- | --- | --- | --- | --- | --- | --- | --- | --- |
| 0 | Total N | | | 196 | | |  |  |  |
|  | Test Statistic | | | .000^a,b^ | | |  |  |  |
|  | Degree Of Freedom | | | 9 | | |  |  |  |
|  | Asymptotic Sig.(2-sided test) | | | 1.000 | | |  |  |  |
| 1 | Total N | | | 204 | | |  |  |  |
|  | Test Statistic | | | 78.576^a^ | | |  |  |  |
|  | Degree Of Freedom | | | 9 | | |  |  |  |
|  | Asymptotic Sig.(2-sided test) | | | .000 | | |  |  |  |
| a. The test statistic is adjusted for ties. | | | | | | |  |  |  |
| b. Multiple comparisons are not performed because the overall test does not show significant differences across samples. | | | | | | |  |  |  |
| **Pairwise Comparisons of Date** | | | | | | |  |  |  |
| Positive | | Sample 1-Sample 2 | Test Statistic | | Std. Error | Std. Test Statistic | | Sig. | Adj. Sig.^a^ |
| 1 | | 6-8.07-26-28.10 | -11.700 | | 31.422 | -.372 | | .710 | 1.000 |
|  |  | 6-8.07-20-22.07 | -11.795 | | 21.185 | -.557 | | .578 | 1.000 |
|  |  | 6-8.07-12-14.10 | -31.900 | | 22.863 | -1.395 | | .163 | 1.000 |
|  |  | 6-8.07-3-5.08 | -43.442 | | 20.602 | -2.109 | | .035 | 1.000 |
|  |  | 6-8.07-22-24.06 | 45.500 | | 38.105 | 1.194 | | .232 | 1.000 |
|  |  | 6-8.07-17-19.08 | -56.107 | | 20.368 | -2.755 | | .006 | .264 |
|  |  | 6-8.07-28-30.09 | -89.355 | | 20.070 | -4.452 | | .000 | .000 |
|  |  | 6-8.07-31.08-2.09 | -97.437 | | 19.983 | -4.876 | | .000 | .000 |
|  |  | 6-8.07-14-16.09 | -111.017 | | 20.163 | -5.506 | | .000 | .000 |
|  |  | 26-28.10-20-22.07 | .095 | | 29.247 | .003 | | .997 | 1.000 |
|  |  | 26-28.10-12-14.10 | 20.200 | | 30.484 | .663 | | .508 | 1.000 |
|  |  | 26-28.10-3-5.08 | 31.742 | | 28.827 | 1.101 | | .271 | 1.000 |
|  |  | 26-28.10-22-24.06 | 33.800 | | 43.111 | .784 | | .433 | 1.000 |
|  |  | 26-28.10-17-19.08 | 44.407 | | 28.660 | 1.549 | | .121 | 1.000 |
|  |  | 26-28.10-28-30.09 | 77.655 | | 28.450 | 2.730 | | .006 | .285 |
|  |  | 26-28.10-31.08-2.09 | 85.738 | | 28.388 | 3.020 | | .003 | .114 |
|  |  | 26-28.10-14-16.09 | 99.317 | | 28.515 | 3.483 | | .000 | .022 |
|  |  | 20-22.07-12-14.10 | -20.105 | | 19.767 | -1.017 | | .309 | 1.000 |
|  |  | 20-22.07-3-5.08 | -31.647 | | 17.101 | -1.851 | | .064 | 1.000 |
|  |  | 20-22.07-22-24.06 | 33.705 | | 36.332 | .928 | | .354 | 1.000 |
|  |  | 20-22.07-17-19.08 | -44.312 | | 16.818 | -2.635 | | .008 | .379 |
|  |  | 20-22.07-28-30.09 | -77.559 | | 16.456 | -4.713 | | .000 | .000 |
|  |  | 20-22.07-31.08-2.09 | -85.642 | | 16.349 | -5.238 | | .000 | .000 |
|  |  | 20-22.07-14-16.09 | -99.221 | | 16.570 | -5.988 | | .000 | .000 |
|  |  | 12-14.10-3-5.08 | 11.542 | | 19.140 | .603 | | .546 | 1.000 |
|  |  | 12-14.10-22-24.06 | 13.600 | | 37.335 | .364 | | .716 | 1.000 |
|  |  | 12-14.10-17-19.08 | 24.207 | | 18.889 | 1.282 | | .200 | 1.000 |
|  |  | 12-14.10-28-30.09 | 57.455 | | 18.567 | 3.094 | | .002 | .089 |
|  |  | 12-14.10-31.08-2.09 | 65.538 | | 18.472 | 3.548 | | .000 | .017 |
|  |  | 12-14.10-14-16.09 | 79.117 | | 18.668 | 4.238 | | .000 | .001 |
|  |  | 3-5.08-22-24.06 | 2.058 | | 35.995 | .057 | | .954 | 1.000 |
|  |  | 3-5.08-17-19.08 | -12.665 | | 16.078 | -.788 | | .431 | 1.000 |
|  |  | 3-5.08-28-30.09 | -45.913 | | 15.699 | -2.925 | | .003 | .155 |
|  |  | 3-5.08-31.08-2.09 | -53.995 | | 15.586 | -3.464 | | .001 | .024 |
|  |  | 3-5.08-14-16.09 | -67.574 | | 15.817 | -4.272 | | .000 | .001 |
|  |  | 22-24.06-17-19.08 | -10.607 | | 35.862 | -.296 | | .767 | 1.000 |
|  |  | 22-24.06-28-30.09 | -43.855 | | 35.693 | -1.229 | | .219 | 1.000 |
|  |  | 22-24.06-31.08-2.09 | -51.937 | | 35.644 | -1.457 | | .145 | 1.000 |
|  |  | 22-24.06-14-16.09 | -65.517 | | 35.746 | -1.833 | | .067 | 1.000 |
|  |  | 17-19.08-28-30.09 | -33.248 | | 15.391 | -2.160 | | .031 | 1.000 |
|  |  | 17-19.08-31.08-2.09 | -41.330 | | 15.276 | -2.706 | | .007 | .307 |
|  |  | 17-19.08-14-16.09 | -54.910 | | 15.512 | -3.540 | | .000 | .018 |
|  |  | 28-30.09-31.08-2.09 | 8.083 | | 14.877 | .543 | | .587 | 1.000 |
|  |  | 28-30.09-14-16.09 | 21.662 | | 15.119 | 1.433 | | .152 | 1.000 |
|  |  | 31.08-2.09-14-16.09 | -13.579 | | 15.002 | -.905 | | .365 | 1.000 |
| Each row tests the null hypothesis that the Sample 1 and Sample 2 distributions are the same.  Asymptotic significances (2-sided tests) are displayed. The significance level is .050. | | | | | | | | | |
| a. Significance values have been adjusted by the Bonferroni correction for multiple tests. | | | | | | | | | |

# Gender

## Prevalence

| **Chi-Square Tests** | | | | | | |
| --- | --- | --- | --- | --- | --- | --- |
| Date | | Value | df | Asymptotic Significance (2-sided) | Exact Sig. (2-sided) | Exact Sig. (1-sided) |
| 28-30.09 | Pearson Chi-Square | .018^a^ | 1 | .893 |  |  |
|  | Continuity Correction^b^ | .000 | 1 | 1.000 |  |  |
|  | Likelihood Ratio | .018 | 1 | .894 |  |  |
|  | Fisher's Exact Test |  |  |  | 1.000 | .594 |
|  | N of Valid Cases | 40 |  |  |  |  |
| 12-14.10 | Pearson Chi-Square | .825^c^ | 1 | .364 |  |  |
|  | Continuity Correction^b^ | .334 | 1 | .563 |  |  |
|  | Likelihood Ratio | .836 | 1 | .361 |  |  |
|  | Fisher's Exact Test |  |  |  | .512 | .283 |
|  | N of Valid Cases | 40 |  |  |  |  |
| 26-28.10 | Pearson Chi-Square | 1.184^d^ | 1 | .277 |  |  |
|  | Continuity Correction^b^ | .365 | 1 | .546 |  |  |
|  | Likelihood Ratio | 1.282 | 1 | .258 |  |  |
|  | Fisher's Exact Test |  |  |  | .373 | .280 |
|  | N of Valid Cases | 40 |  |  |  |  |
| 22-24.06 | Pearson Chi-Square | 2.397^e^ | 1 | .122 |  |  |
|  | Continuity Correction^b^ | .886 | 1 | .347 |  |  |
|  | Likelihood Ratio | 3.499 | 1 | .061 |  |  |
|  | Fisher's Exact Test |  |  |  | .248 | .179 |
|  | N of Valid Cases | 40 |  |  |  |  |
| 6-8.07 | Pearson Chi-Square | .395^f^ | 1 | .530 |  |  |
|  | Continuity Correction^b^ | .078 | 1 | .780 |  |  |
|  | Likelihood Ratio | .392 | 1 | .531 |  |  |
|  | Fisher's Exact Test |  |  |  | .728 | .388 |
|  | N of Valid Cases | 40 |  |  |  |  |
| 20-22.07 | Pearson Chi-Square | .753^g^ | 1 | .385 |  |  |
|  | Continuity Correction^b^ | .299 | 1 | .585 |  |  |
|  | Likelihood Ratio | .754 | 1 | .385 |  |  |
|  | Fisher's Exact Test |  |  |  | .523 | .292 |
|  | N of Valid Cases | 40 |  |  |  |  |
| 3-5.08 | Pearson Chi-Square | .001^h^ | 1 | .973 |  |  |
|  | Continuity Correction^b^ | .000 | 1 | 1.000 |  |  |
|  | Likelihood Ratio | .001 | 1 | .973 |  |  |
|  | Fisher's Exact Test |  |  |  | 1.000 | .616 |
|  | N of Valid Cases | 40 |  |  |  |  |
| 17-19.08 | Pearson Chi-Square | 1.759^f^ | 1 | .185 |  |  |
|  | Continuity Correction^b^ | .955 | 1 | .328 |  |  |
|  | Likelihood Ratio | 1.749 | 1 | .186 |  |  |
|  | Fisher's Exact Test |  |  |  | .296 | .164 |
|  | N of Valid Cases | 40 |  |  |  |  |
| 31.08-2.09 | Pearson Chi-Square | .230^i^ | 1 | .631 |  |  |
|  | Continuity Correction^b^ | .006 | 1 | .936 |  |  |
|  | Likelihood Ratio | .228 | 1 | .633 |  |  |
|  | Fisher's Exact Test |  |  |  | .702 | .463 |
|  | N of Valid Cases | 40 |  |  |  |  |
| 14-16.09 | Pearson Chi-Square | 1.671^j^ | 1 | .196 |  |  |
|  | Continuity Correction^b^ | .853 | 1 | .356 |  |  |
|  | Likelihood Ratio | 1.659 | 1 | .198 |  |  |
|  | Fisher's Exact Test |  |  |  | .274 | .178 |
|  | N of Valid Cases | 40 |  |  |  |  |
| Total | Pearson Chi-Square | 1.687^k^ | 1 | .194 |  |  |
|  | Continuity Correction^b^ | .724 | 1 | .395 |  |  |
|  | Likelihood Ratio | 1.676 | 1 | .195 |  |  |
|  | Fisher's Exact Test |  |  |  | .373 | .197 |
|  | N of Valid Cases | 40 |  |  |  |  |
| a. 1 cells (25.0%) have expected count less than 5. The minimum expected count is 3.83. | | | | | | |
| b. Computed only for a 2x2 table | | | | | | |
| c. 0 cells (0.0%) have expected count less than 5. The minimum expected count is 6.38. | | | | | | |
| d. 2 cells (50.0%) have expected count less than 5. The minimum expected count is 2.13. | | | | | | |
| e. 2 cells (50.0%) have expected count less than 5. The minimum expected count is 1.27. | | | | | | |
| f. 0 cells (0.0%) have expected count less than 5. The minimum expected count is 5.10. | | | | | | |
| g. 0 cells (0.0%) have expected count less than 5. The minimum expected count is 7.65. | | | | | | |
| h. 0 cells (0.0%) have expected count less than 5. The minimum expected count is 5.95. | | | | | | |
| i. 2 cells (50.0%) have expected count less than 5. The minimum expected count is 3.40. | | | | | | |
| j. 1 cells (25.0%) have expected count less than 5. The minimum expected count is 4.25. | | | | | | |
| k. 2 cells (50.0%) have expected count less than 5. The minimum expected count is 2.55. | | | | | | |

## Mean Abundance

|  |
| --- |

| **Independent-Samples Mann-Whitney U Test Summary** | | |
| --- | --- | --- |
| 28-30.09 | Total N | 40 |
|  | Mann-Whitney U | 173.000 |
|  | Wilcoxon W | 326.000 |
|  | Test Statistic | 173.000 |
|  | Standard Error | 36.344 |
|  | Standardized Test Statistic | -.619 |
|  | Asymptotic Sig.(2-sided test) | .536 |
|  | Exact Sig.(2-sided test) | .551 |
| 12-14.10 | Total N | 40 |
|  | Mann-Whitney U | 153.000 |
|  | Wilcoxon W | 306.000 |
|  | Test Statistic | 153.000 |
|  | Standard Error | 31.780 |
|  | Standardized Test Statistic | -1.337 |
|  | Asymptotic Sig.(2-sided test) | .181 |
|  | Exact Sig.(2-sided test) | .254 |
| 26-28.10 | Total N | 40 |
|  | Mann-Whitney U | 172.000 |
|  | Wilcoxon W | 325.000 |
|  | Test Statistic | 172.000 |
|  | Standard Error | 21.003 |
|  | Standardized Test Statistic | -1.119 |
|  | Asymptotic Sig.(2-sided test) | .263 |
|  | Exact Sig.(2-sided test) | .533 |
| 22-24.06 | Total N | 40 |
|  | Mann-Whitney U | 170.000 |
|  | Wilcoxon W | 323.000 |
|  | Test Statistic | 170.000 |
|  | Standard Error | 16.695 |
|  | Standardized Test Statistic | -1.527 |
|  | Asymptotic Sig.(2-sided test) | .127 |
|  | Exact Sig.(2-sided test) | .498 |
| 6-8.07 | Total N | 40 |
|  | Mann-Whitney U | 211.500 |
|  | Wilcoxon W | 364.500 |
|  | Test Statistic | 211.500 |
|  | Standard Error | 29.629 |
|  | Standardized Test Statistic | .540 |
|  | Asymptotic Sig.(2-sided test) | .589 |
|  | Exact Sig.(2-sided test) | .665 |
| 20-22.07 | Total N | 40 |
|  | Mann-Whitney U | 158.000 |
|  | Wilcoxon W | 311.000 |
|  | Test Statistic | 158.000 |
|  | Standard Error | 34.848 |
|  | Standardized Test Statistic | -1.076 |
|  | Asymptotic Sig.(2-sided test) | .282 |
|  | Exact Sig.(2-sided test) | .315 |
| 3-5.08 | Total N | 40 |
|  | Mann-Whitney U | 188.500 |
|  | Wilcoxon W | 341.500 |
|  | Test Statistic | 188.500 |
|  | Standard Error | 35.760 |
|  | Standardized Test Statistic | -.196 |
|  | Asymptotic Sig.(2-sided test) | .845 |
|  | Exact Sig.(2-sided test) | .850 |
| 17-19.08 | Total N | 40 |
|  | Mann-Whitney U | 164.500 |
|  | Wilcoxon W | 317.500 |
|  | Test Statistic | 164.500 |
|  | Standard Error | 36.055 |
|  | Standardized Test Statistic | -.860 |
|  | Asymptotic Sig.(2-sided test) | .390 |
|  | Exact Sig.(2-sided test) | .401 |
| 31.08-2.09 | Total N | 40 |
|  | Mann-Whitney U | 190.000 |
|  | Wilcoxon W | 343.000 |
|  | Test Statistic | 190.000 |
|  | Standard Error | 36.406 |
|  | Standardized Test Statistic | -.151 |
|  | Asymptotic Sig.(2-sided test) | .880 |
|  | Exact Sig.(2-sided test) | .892 |
| 14-16.09 | Total N | 40 |
|  | Mann-Whitney U | 150.000 |
|  | Wilcoxon W | 303.000 |
|  | Test Statistic | 150.000 |
|  | Standard Error | 36.264 |
|  | Standardized Test Statistic | -1.255 |
|  | Asymptotic Sig.(2-sided test) | .210 |
|  | Exact Sig.(2-sided test) | .221 |
| Total | Total N | 40 |
|  | Mann-Whitney U | 164.000 |
|  | Wilcoxon W | 317.000 |
|  | Test Statistic | 164.000 |
|  | Standard Error | 36.490 |
|  | Standardized Test Statistic | -.863 |
|  | Asymptotic Sig.(2-sided test) | .388 |
|  | Exact Sig.(2-sided test) | .401 |

## Mean intensity

| **Hypothesis Test Summary** | | | | | | |
| --- | --- | --- | --- | --- | --- | --- |
| Date | Positive | | Null Hypothesis | Test | Sig.^a,b^ | Decision |
| 28-30.09 | 0 | 1 | The distribution of Total is the same across categories of Gender. | Independent-Samples Mann-Whitney U Test | 1.000^c^ | Retain the null hypothesis. |
|  | 1 | 1 | The distribution of Total is the same across categories of Gender. | Independent-Samples Mann-Whitney U Test | .465^c^ | Retain the null hypothesis. |
| 12-14.10 | 0 | 1 | The distribution of Total is the same across categories of Gender. | Independent-Samples Mann-Whitney U Test | 1.000^c^ | Retain the null hypothesis. |
|  | 1 | 1 | The distribution of Total is the same across categories of Gender. | Independent-Samples Mann-Whitney U Test | .075^c^ | Retain the null hypothesis. |
| 26-28.10 | 0 | 1 | The distribution of Total is the same across categories of Gender. | Independent-Samples Mann-Whitney U Test | 1.000^c^ | Retain the null hypothesis. |
|  | 1 | 1 | The distribution of Total is the same across categories of Gender. | Independent-Samples Mann-Whitney U Test | .800^c^ | Retain the null hypothesis. |
| 22-24.06 | 0 | 1 | The distribution of Total is the same across categories of Gender. | Independent-Samples Mann-Whitney U Test | 1.000^c^ | Retain the null hypothesis. |
| 6-8.07 | 0 | 1 | The distribution of Total is the same across categories of Gender. | Independent-Samples Mann-Whitney U Test | 1.000^c^ | Retain the null hypothesis. |
|  | 1 | 1 | The distribution of Total is the same across categories of Gender. | Independent-Samples Mann-Whitney U Test | .818^c^ | Retain the null hypothesis. |
| 20-22.07 | 0 | 1 | The distribution of Total is the same across categories of Gender. | Independent-Samples Mann-Whitney U Test | 1.000^c^ | Retain the null hypothesis. |
|  | 1 | 1 | The distribution of Total is the same across categories of Gender. | Independent-Samples Mann-Whitney U Test | .482^c^ | Retain the null hypothesis. |
| 3-5.08 | 0 | 1 | The distribution of Total is the same across categories of Gender. | Independent-Samples Mann-Whitney U Test | 1.000^c^ | Retain the null hypothesis. |
|  | 1 | 1 | The distribution of Total is the same across categories of Gender. | Independent-Samples Mann-Whitney U Test | .760^c^ | Retain the null hypothesis. |
| 17-19.08 | 0 | 1 | The distribution of Total is the same across categories of Gender. | Independent-Samples Mann-Whitney U Test | 1.000^c^ | Retain the null hypothesis. |
|  | 1 | 1 | The distribution of Total is the same across categories of Gender. | Independent-Samples Mann-Whitney U Test | .759^c^ | Retain the null hypothesis. |
| 31.08-2.09 | 0 | 1 | The distribution of Total is the same across categories of Gender. | Independent-Samples Mann-Whitney U Test | 1.000^c^ | Retain the null hypothesis. |
|  | 1 | 1 | The distribution of Total is the same across categories of Gender. | Independent-Samples Mann-Whitney U Test | .821^c^ | Retain the null hypothesis. |
| 14-16.09 | 0 | 1 | The distribution of Total is the same across categories of Gender. | Independent-Samples Mann-Whitney U Test | 1.000^c^ | Retain the null hypothesis. |
|  | 1 | 1 | The distribution of Total is the same across categories of Gender. | Independent-Samples Mann-Whitney U Test | .672^c^ | Retain the null hypothesis. |
| Total | 0 | 1 | The distribution of Total is the same across categories of Gender. | Independent-Samples Mann-Whitney U Test | 1.000^c^ | Retain the null hypothesis. |
|  | 1 | 1 | The distribution of Total is the same across categories of Gender. | Independent-Samples Mann-Whitney U Test | .944^c^ | Retain the null hypothesis. |
| a. The significance level is .050. | | | | | | |
| b. Asymptotic significance is displayed. | | | | | | |
| c. Exact significance is displayed for this test. | | | | | | |

**Independent-Samples Mann-Whitney U Test**

**Total across Gender**

| **Independent-Samples Mann-Whitney U Test Summary** | | | |
| --- | --- | --- | --- |
| 28-30.09 | 0 | Total N | 9 |
|  |  | Mann-Whitney U | 10.000 |
|  |  | Wilcoxon W | 20.000 |
|  |  | Test Statistic | 10.000 |
|  |  | Standard Error | .000 |
|  |  | Standardized Test Statistic | .000 |
|  |  | Asymptotic Sig.(2-sided test) | 1.000 |
|  |  | Exact Sig.(2-sided test) | 1.000 |
|  | 1 | Total N | 31 |
|  |  | Mann-Whitney U | 98.000 |
|  |  | Wilcoxon W | 189.000 |
|  |  | Test Statistic | 98.000 |
|  |  | Standard Error | 24.980 |
|  |  | Standardized Test Statistic | -.761 |
|  |  | Asymptotic Sig.(2-sided test) | .447 |
|  |  | Exact Sig.(2-sided test) | .465 |
| 12-14.10 | 0 | Total N | 25 |
|  |  | Mann-Whitney U | 78.000 |
|  |  | Wilcoxon W | 156.000 |
|  |  | Test Statistic | 78.000 |
|  |  | Standard Error | .000 |
|  |  | Standardized Test Statistic | .000 |
|  |  | Asymptotic Sig.(2-sided test) | 1.000 |
|  |  | Exact Sig.(2-sided test) | 1.000 |
|  | 1 | Total N | 15 |
|  |  | Mann-Whitney U | 10.000 |
|  |  | Wilcoxon W | 25.000 |
|  |  | Test Statistic | 10.000 |
|  |  | Standard Error | 8.158 |
|  |  | Standardized Test Statistic | -1.839 |
|  |  | Asymptotic Sig.(2-sided test) | .066 |
|  |  | Exact Sig.(2-sided test) | .075 |
| 26-28.10 | 0 | Total N | 35 |
|  |  | Mann-Whitney U | 152.000 |
|  |  | Wilcoxon W | 288.000 |
|  |  | Test Statistic | 152.000 |
|  |  | Standard Error | .000 |
|  |  | Standardized Test Statistic | .000 |
|  |  | Asymptotic Sig.(2-sided test) | 1.000 |
|  |  | Exact Sig.(2-sided test) | 1.000 |
|  | 1 | Total N | 5 |
|  |  | Mann-Whitney U | 1.000 |
|  |  | Wilcoxon W | 2.000 |
|  |  | Test Statistic | 1.000 |
|  |  | Standard Error | 1.414 |
|  |  | Standardized Test Statistic | -.707 |
|  |  | Asymptotic Sig.(2-sided test) | .480 |
|  |  | Exact Sig.(2-sided test) | .800 |
| 22-24.06 | 0 | Total N | 37 |
|  |  | Mann-Whitney U | 170.000 |
|  |  | Wilcoxon W | 323.000 |
|  |  | Test Statistic | 170.000 |
|  |  | Standard Error | .000 |
|  |  | Standardized Test Statistic | .000 |
|  |  | Asymptotic Sig.(2-sided test) | 1.000 |
|  |  | Exact Sig.(2-sided test) | 1.000 |
| 6-8.07 | 0 | Total N | 28 |
|  |  | Mann-Whitney U | 93.500 |
|  |  | Wilcoxon W | 159.500 |
|  |  | Test Statistic | 93.500 |
|  |  | Standard Error | .000 |
|  |  | Standardized Test Statistic | .000 |
|  |  | Asymptotic Sig.(2-sided test) | 1.000 |
|  |  | Exact Sig.(2-sided test) | 1.000 |
|  | 1 | Total N | 12 |
|  |  | Mann-Whitney U | 16.000 |
|  |  | Wilcoxon W | 37.000 |
|  |  | Test Statistic | 16.000 |
|  |  | Standard Error | 6.234 |
|  |  | Standardized Test Statistic | -.321 |
|  |  | Asymptotic Sig.(2-sided test) | .748 |
|  |  | Exact Sig.(2-sided test) | .818 |
| 20-22.07 | 0 | Total N | 18 |
|  |  | Mann-Whitney U | 40.500 |
|  |  | Wilcoxon W | 85.500 |
|  |  | Test Statistic | 40.500 |
|  |  | Standard Error | .000 |
|  |  | Standardized Test Statistic | .000 |
|  |  | Asymptotic Sig.(2-sided test) | 1.000 |
|  |  | Exact Sig.(2-sided test) | 1.000 |
|  | 1 | Total N | 22 |
|  |  | Mann-Whitney U | 45.500 |
|  |  | Wilcoxon W | 81.500 |
|  |  | Test Statistic | 45.500 |
|  |  | Standard Error | 14.647 |
|  |  | Standardized Test Statistic | -.717 |
|  |  | Asymptotic Sig.(2-sided test) | .473 |
|  |  | Exact Sig.(2-sided test) | .482 |
| 3-5.08 | 0 | Total N | 14 |
|  |  | Mann-Whitney U | 24.000 |
|  |  | Wilcoxon W | 45.000 |
|  |  | Test Statistic | 24.000 |
|  |  | Standard Error | .000 |
|  |  | Standardized Test Statistic | .000 |
|  |  | Asymptotic Sig.(2-sided test) | 1.000 |
|  |  | Exact Sig.(2-sided test) | 1.000 |
|  | 1 | Total N | 26 |
|  |  | Mann-Whitney U | 76.500 |
|  |  | Wilcoxon W | 142.500 |
|  |  | Test Statistic | 76.500 |
|  |  | Standard Error | 19.265 |
|  |  | Standardized Test Statistic | -.311 |
|  |  | Asymptotic Sig.(2-sided test) | .755 |
|  |  | Exact Sig.(2-sided test) | .760 |
| 17-19.08 | 0 | Total N | 12 |
|  |  | Mann-Whitney U | 17.500 |
|  |  | Wilcoxon W | 45.500 |
|  |  | Test Statistic | 17.500 |
|  |  | Standard Error | .000 |
|  |  | Standardized Test Statistic | .000 |
|  |  | Asymptotic Sig.(2-sided test) | 1.000 |
|  |  | Exact Sig.(2-sided test) | 1.000 |
|  | 1 | Total N | 28 |
|  |  | Mann-Whitney U | 97.000 |
|  |  | Wilcoxon W | 152.000 |
|  |  | Test Statistic | 97.000 |
|  |  | Standard Error | 20.854 |
|  |  | Standardized Test Statistic | .336 |
|  |  | Asymptotic Sig.(2-sided test) | .737 |
|  |  | Exact Sig.(2-sided test) | .759 |
| 31.08-2.09 | 0 | Total N | 8 |
|  |  | Mann-Whitney U | 8.000 |
|  |  | Wilcoxon W | 18.000 |
|  |  | Test Statistic | 8.000 |
|  |  | Standard Error | .000 |
|  |  | Standardized Test Statistic | .000 |
|  |  | Asymptotic Sig.(2-sided test) | 1.000 |
|  |  | Exact Sig.(2-sided test) | 1.000 |
|  | 1 | Total N | 32 |
|  |  | Mann-Whitney U | 130.000 |
|  |  | Wilcoxon W | 221.000 |
|  |  | Test Statistic | 130.000 |
|  |  | Standard Error | 26.062 |
|  |  | Standardized Test Statistic | .249 |
|  |  | Asymptotic Sig.(2-sided test) | .803 |
|  |  | Exact Sig.(2-sided test) | .821 |
| 14-16.09 | 0 | Total N | 10 |
|  |  | Mann-Whitney U | 12.000 |
|  |  | Wilcoxon W | 33.000 |
|  |  | Test Statistic | 12.000 |
|  |  | Standard Error | .000 |
|  |  | Standardized Test Statistic | .000 |
|  |  | Asymptotic Sig.(2-sided test) | 1.000 |
|  |  | Exact Sig.(2-sided test) | 1.000 |
|  | 1 | Total N | 30 |
|  |  | Mann-Whitney U | 94.000 |
|  |  | Wilcoxon W | 160.000 |
|  |  | Test Statistic | 94.000 |
|  |  | Standard Error | 23.234 |
|  |  | Standardized Test Statistic | -.452 |
|  |  | Asymptotic Sig.(2-sided test) | .651 |
|  |  | Exact Sig.(2-sided test) | .672 |
| Total | 0 | Total N | 6 |
|  |  | Mann-Whitney U | 4.000 |
|  |  | Wilcoxon W | 14.000 |
|  |  | Test Statistic | 4.000 |
|  |  | Standard Error | .000 |
|  |  | Standardized Test Statistic | .000 |
|  |  | Asymptotic Sig.(2-sided test) | 1.000 |
|  |  | Exact Sig.(2-sided test) | 1.000 |
|  | 1 | Total N | 34 |
|  |  | Mann-Whitney U | 134.000 |
|  |  | Wilcoxon W | 225.000 |
|  |  | Test Statistic | 134.000 |
|  |  | Standard Error | 28.218 |
|  |  | Standardized Test Statistic | -.089 |
|  |  | Asymptotic Sig.(2-sided test) | .929 |
|  |  | Exact Sig.(2-sided test) | .944 |

# Age Group

## Prevalence

| **Crosstab** | | | | | | |
| --- | --- | --- | --- | --- | --- | --- |
| Count | | | | | | |
| Date | | | AgeGroup | | | Total |
|  |  |  | 1.00 | 2.00 | 3.00 |  |
| 28-30.09 | Positive | 0 | 0 | 6 | 3 | 9 |
|  |  | 1 | 6 | 11 | 14 | 31 |
|  | Total | | 6 | 17 | 17 | 40 |
| 12-14.10 | Positive | 0 | 2 | 10 | 13 | 25 |
|  |  | 1 | 4 | 7 | 4 | 15 |
|  | Total | | 6 | 17 | 17 | 40 |
| 26-28.10 | Positive | 0 | 5 | 13 | 17 | 35 |
|  |  | 1 | 1 | 4 | 0 | 5 |
|  | Total | | 6 | 17 | 17 | 40 |
| 22-24.06 | Positive | 0 | 6 | 14 | 17 | 37 |
|  |  | 1 | 0 | 3 | 0 | 3 |
|  | Total | | 6 | 17 | 17 | 40 |
| 6-8.07 | Positive | 0 | 4 | 14 | 10 | 28 |
|  |  | 1 | 2 | 3 | 7 | 12 |
|  | Total | | 6 | 17 | 17 | 40 |
| 20-22.07 | Positive | 0 | 3 | 8 | 7 | 18 |
|  |  | 1 | 3 | 9 | 10 | 22 |
|  | Total | | 6 | 17 | 17 | 40 |
| 3-5.08 | Positive | 0 | 1 | 7 | 6 | 14 |
|  |  | 1 | 5 | 10 | 11 | 26 |
|  | Total | | 6 | 17 | 17 | 40 |
| 17-19.08 | Positive | 0 | 2 | 6 | 4 | 12 |
|  |  | 1 | 4 | 11 | 13 | 28 |
|  | Total | | 6 | 17 | 17 | 40 |
| 31.08-2.09 | Positive | 0 | 0 | 4 | 4 | 8 |
|  |  | 1 | 6 | 13 | 13 | 32 |
|  | Total | | 6 | 17 | 17 | 40 |
| 14-16.09 | Positive | 0 | 1 | 5 | 4 | 10 |
|  |  | 1 | 5 | 12 | 13 | 30 |
|  | Total | | 6 | 17 | 17 | 40 |
| Total | Positive | 0 | 0 | 3 | 3 | 6 |
|  |  | 1 | 6 | 14 | 14 | 34 |
|  | Total | | 6 | 17 | 17 | 40 |

| **Chi-Square Tests** | | | | |
| --- | --- | --- | --- | --- |
| Date | | Value | df | Asymptotic Significance (2-sided) |
| 28-30.09 | Pearson Chi-Square | 3.567^a^ | 2 | .168 |
|  | Likelihood Ratio | 4.735 | 2 | .094 |
|  | Linear-by-Linear Association | .077 | 1 | .781 |
|  | N of Valid Cases | 40 |  |  |
| 12-14.10 | Pearson Chi-Square | 3.692^b^ | 2 | .158 |
|  | Likelihood Ratio | 3.702 | 2 | .157 |
|  | Linear-by-Linear Association | 3.544 | 1 | .060 |
|  | N of Valid Cases | 40 |  |  |
| 26-28.10 | Pearson Chi-Square | 4.415^c^ | 2 | .110 |
|  | Likelihood Ratio | 6.185 | 2 | .045 |
|  | Linear-by-Linear Association | 2.517 | 1 | .113 |
|  | N of Valid Cases | 40 |  |  |
| 22-24.06 | Pearson Chi-Square | 4.388^d^ | 2 | .111 |
|  | Likelihood Ratio | 5.467 | 2 | .065 |
|  | Linear-by-Linear Association | .479 | 1 | .489 |
|  | N of Valid Cases | 40 |  |  |
| 6-8.07 | Pearson Chi-Square | 2.278^e^ | 2 | .320 |
|  | Likelihood Ratio | 2.352 | 2 | .308 |
|  | Linear-by-Linear Association | .672 | 1 | .412 |
|  | N of Valid Cases | 40 |  |  |
| 20-22.07 | Pearson Chi-Square | .190^f^ | 2 | .909 |
|  | Likelihood Ratio | .190 | 2 | .909 |
|  | Linear-by-Linear Association | .178 | 1 | .673 |
|  | N of Valid Cases | 40 |  |  |
| 3-5.08 | Pearson Chi-Square | 1.172^g^ | 2 | .557 |
|  | Likelihood Ratio | 1.280 | 2 | .527 |
|  | Linear-by-Linear Association | .284 | 1 | .594 |
|  | N of Valid Cases | 40 |  |  |
| 17-19.08 | Pearson Chi-Square | .598^e^ | 2 | .742 |
|  | Likelihood Ratio | .606 | 2 | .738 |
|  | Linear-by-Linear Association | .393 | 1 | .531 |
|  | N of Valid Cases | 40 |  |  |
| 31.08-2.09 | Pearson Chi-Square | 1.765^h^ | 2 | .414 |
|  | Likelihood Ratio | 2.932 | 2 | .231 |
|  | Linear-by-Linear Association | .988 | 1 | .320 |
|  | N of Valid Cases | 40 |  |  |
| 14-16.09 | Pearson Chi-Square | .418^i^ | 2 | .811 |
|  | Likelihood Ratio | .433 | 2 | .805 |
|  | Linear-by-Linear Association | .016 | 1 | .899 |
|  | N of Valid Cases | 40 |  |  |
| Total | Pearson Chi-Square | 1.246^j^ | 2 | .536 |
|  | Likelihood Ratio | 2.129 | 2 | .345 |
|  | Linear-by-Linear Association | .698 | 1 | .404 |
|  | N of Valid Cases | 40 |  |  |
| a. 4 cells (66.7%) have expected count less than 5. The minimum expected count is 1.35. | | | | |
| b. 2 cells (33.3%) have expected count less than 5. The minimum expected count is 2.25. | | | | |
| c. 3 cells (50.0%) have expected count less than 5. The minimum expected count is .75. | | | | |
| d. 3 cells (50.0%) have expected count less than 5. The minimum expected count is .45. | | | | |
| e. 2 cells (33.3%) have expected count less than 5. The minimum expected count is 1.80. | | | | |
| f. 2 cells (33.3%) have expected count less than 5. The minimum expected count is 2.70. | | | | |
| g. 2 cells (33.3%) have expected count less than 5. The minimum expected count is 2.10. | | | | |
| h. 4 cells (66.7%) have expected count less than 5. The minimum expected count is 1.20. | | | | |
| i. 4 cells (66.7%) have expected count less than 5. The minimum expected count is 1.50. | | | | |
| j. 3 cells (50.0%) have expected count less than 5. The minimum expected count is .90. | | | | |

## Mean Abundance

| **Independent-Samples Kruskal-Wallis Test Summary** | | |
| --- | --- | --- |
| 28-30.09 | Total N | 40 |
|  | Test Statistic | 1.100^a,b^ |
|  | Degree Of Freedom | 2 |
|  | Asymptotic Sig.(2-sided test) | .577 |
| 12-14.10 | Total N | 40 |
|  | Test Statistic | 5.900^a,b^ |
|  | Degree Of Freedom | 2 |
|  | Asymptotic Sig.(2-sided test) | .052 |
| 26-28.10 | Total N | 40 |
|  | Test Statistic | 4.346^a,b^ |
|  | Degree Of Freedom | 2 |
|  | Asymptotic Sig.(2-sided test) | .114 |
| 22-24.06 | Total N | 40 |
|  | Test Statistic | 4.271^a,b^ |
|  | Degree Of Freedom | 2 |
|  | Asymptotic Sig.(2-sided test) | .118 |
| 6-8.07 | Total N | 40 |
|  | Test Statistic | 3.900^a,b^ |
|  | Degree Of Freedom | 2 |
|  | Asymptotic Sig.(2-sided test) | .142 |
| 20-22.07 | Total N | 40 |
|  | Test Statistic | .429^a,b^ |
|  | Degree Of Freedom | 2 |
|  | Asymptotic Sig.(2-sided test) | .807 |
| 3-5.08 | Total N | 40 |
|  | Test Statistic | 5.126^a,b^ |
|  | Degree Of Freedom | 2 |
|  | Asymptotic Sig.(2-sided test) | .077 |
| 17-19.08 | Total N | 40 |
|  | Test Statistic | .346^a,b^ |
|  | Degree Of Freedom | 2 |
|  | Asymptotic Sig.(2-sided test) | .841 |
| 31.08-2.09 | Total N | 40 |
|  | Test Statistic | 2.150^a,b^ |
|  | Degree Of Freedom | 2 |
|  | Asymptotic Sig.(2-sided test) | .341 |
| 14-16.09 | Total N | 40 |
|  | Test Statistic | .834^a,b^ |
|  | Degree Of Freedom | 2 |
|  | Asymptotic Sig.(2-sided test) | .659 |
| Total | Total N | 40 |
|  | Test Statistic | 1.505^a,b^ |
|  | Degree Of Freedom | 2 |
|  | Asymptotic Sig.(2-sided test) | .471 |
| a. The test statistic is adjusted for ties. | | |
| b. Multiple comparisons are not performed because the overall test does not show significant differences across samples. | | |

## Mean intensity

| **Hypothesis Test Summary** | | | | | | |
| --- | --- | --- | --- | --- | --- | --- |
| Date | Positive | | Null Hypothesis | Test | Sig.^a,b^ | Decision |
| 28-30.09 | 0 | 1 | The distribution of Total is the same across categories of AgeGroup. | Independent-Samples Mann-Whitney U Test | 1.000^c^ | Retain the null hypothesis. |
|  | 1 | 1 | The distribution of Total is the same across categories of AgeGroup. | Independent-Samples Kruskal-Wallis Test | .595 | Retain the null hypothesis. |
| 12-14.10 | 0 | 1 | The distribution of Total is the same across categories of AgeGroup. | Independent-Samples Kruskal-Wallis Test | 1.000 | Retain the null hypothesis. |
|  | 1 | 1 | The distribution of Total is the same across categories of AgeGroup. | Independent-Samples Kruskal-Wallis Test | .064 | Retain the null hypothesis. |
| 26-28.10 | 0 | 1 | The distribution of Total is the same across categories of AgeGroup. | Independent-Samples Kruskal-Wallis Test | 1.000 | Retain the null hypothesis. |
|  | 1 | 1 | The distribution of Total is the same across categories of AgeGroup. | Independent-Samples Mann-Whitney U Test | 1.000^c^ | Retain the null hypothesis. |
| 22-24.06 | 0 | 1 | The distribution of Total is the same across categories of AgeGroup. | Independent-Samples Kruskal-Wallis Test | 1.000 | Retain the null hypothesis. |
| 6-8.07 | 0 | 1 | The distribution of Total is the same across categories of AgeGroup. | Independent-Samples Kruskal-Wallis Test | 1.000 | Retain the null hypothesis. |
|  | 1 | 1 | The distribution of Total is the same across categories of AgeGroup. | Independent-Samples Kruskal-Wallis Test | .024 | Reject the null hypothesis. |
| 20-22.07 | 0 | 1 | The distribution of Total is the same across categories of AgeGroup. | Independent-Samples Kruskal-Wallis Test | 1.000 | Retain the null hypothesis. |
|  | 1 | 1 | The distribution of Total is the same across categories of AgeGroup. | Independent-Samples Kruskal-Wallis Test | .307 | Retain the null hypothesis. |
| 3-5.08 | 0 | 1 | The distribution of Total is the same across categories of AgeGroup. | Independent-Samples Kruskal-Wallis Test | 1.000 | Retain the null hypothesis. |
|  | 1 | 1 | The distribution of Total is the same across categories of AgeGroup. | Independent-Samples Kruskal-Wallis Test | .029 | Reject the null hypothesis. |
| 17-19.08 | 0 | 1 | The distribution of Total is the same across categories of AgeGroup. | Independent-Samples Kruskal-Wallis Test | 1.000 | Retain the null hypothesis. |
|  | 1 | 1 | The distribution of Total is the same across categories of AgeGroup. | Independent-Samples Kruskal-Wallis Test | .747 | Retain the null hypothesis. |
| 31.08-2.09 | 0 | 1 | The distribution of Total is the same across categories of AgeGroup. | Independent-Samples Mann-Whitney U Test | 1.000^c^ | Retain the null hypothesis. |
|  | 1 | 1 | The distribution of Total is the same across categories of AgeGroup. | Independent-Samples Kruskal-Wallis Test | .692 | Retain the null hypothesis. |
| 14-16.09 | 0 | 1 | The distribution of Total is the same across categories of AgeGroup. | Independent-Samples Kruskal-Wallis Test | 1.000 | Retain the null hypothesis. |
|  | 1 | 1 | The distribution of Total is the same across categories of AgeGroup. | Independent-Samples Kruskal-Wallis Test | .589 | Retain the null hypothesis. |
| Total | 0 | 1 | The distribution of Total is the same across categories of AgeGroup. | Independent-Samples Mann-Whitney U Test | 1.000^c^ | Retain the null hypothesis. |
|  | 1 | 1 | The distribution of Total is the same across categories of AgeGroup. | Independent-Samples Kruskal-Wallis Test | .798 | Retain the null hypothesis. |
| a. The significance level is .050. | | | | | | |
| b. Asymptotic significance is displayed. | | | | | | |
| c. Exact significance is displayed for this test. | | | | | | |

# Colour Group

## Prevalence

| **Crosstab** | | | | | |
| --- | --- | --- | --- | --- | --- |
| Count | | | | | |
| Date | | | Colour | | Total |
|  |  |  | Light | Dark |  |
| 28-30.09 | Positive | 0 | 1 | 8 | 9 |
|  |  | 1 | 11 | 20 | 31 |
|  | Total | | 12 | 28 | 40 |
| 12-14.10 | Positive | 0 | 10 | 15 | 25 |
|  |  | 1 | 2 | 13 | 15 |
|  | Total | | 12 | 28 | 40 |
| 26-28.10 | Positive | 0 | 12 | 23 | 35 |
|  |  | 1 | 0 | 5 | 5 |
|  | Total | | 12 | 28 | 40 |
| 22-24.06 | Positive | 0 | 11 | 26 | 37 |
|  |  | 1 | 1 | 2 | 3 |
|  | Total | | 12 | 28 | 40 |
| 6-8.07 | Positive | 0 | 6 | 22 | 28 |
|  |  | 1 | 6 | 6 | 12 |
|  | Total | | 12 | 28 | 40 |
| 20-22.07 | Positive | 0 | 5 | 13 | 18 |
|  |  | 1 | 7 | 15 | 22 |
|  | Total | | 12 | 28 | 40 |
| 3-5.08 | Positive | 0 | 2 | 12 | 14 |
|  |  | 1 | 10 | 16 | 26 |
|  | Total | | 12 | 28 | 40 |
| 17-19.08 | Positive | 0 | 1 | 11 | 12 |
|  |  | 1 | 11 | 17 | 28 |
|  | Total | | 12 | 28 | 40 |
| 31.08-2.09 | Positive | 0 | 2 | 6 | 8 |
|  |  | 1 | 10 | 22 | 32 |
|  | Total | | 12 | 28 | 40 |
| 14-16.09 | Positive | 0 | 2 | 8 | 10 |
|  |  | 1 | 10 | 20 | 30 |
|  | Total | | 12 | 28 | 40 |
| Total | Positive | 0 | 1 | 5 | 6 |
|  |  | 1 | 11 | 23 | 34 |
|  | Total | | 12 | 28 | 40 |

| **Chi-Square Tests** | | | | | | |
| --- | --- | --- | --- | --- | --- | --- |
| Date | | Value | df | Asymptotic Significance (2-sided) | Exact Sig. (2-sided) | Exact Sig. (1-sided) |
| 28-30.09 | Pearson Chi-Square | 1.973^a^ | 1 | .160 |  |  |
|  | Continuity Correction^b^ | .983 | 1 | .321 |  |  |
|  | Likelihood Ratio | 2.266 | 1 | .132 |  |  |
|  | Fisher's Exact Test |  |  |  | .233 | .162 |
|  | N of Valid Cases | 40 |  |  |  |  |
| 12-14.10 | Pearson Chi-Square | 3.175^c^ | 1 | .075 |  |  |
|  | Continuity Correction^b^ | 2.032 | 1 | .154 |  |  |
|  | Likelihood Ratio | 3.438 | 1 | .064 |  |  |
|  | Fisher's Exact Test |  |  |  | .152 | .074 |
|  | N of Valid Cases | 40 |  |  |  |  |
| 26-28.10 | Pearson Chi-Square | 2.449^d^ | 1 | .118 |  |  |
|  | Continuity Correction^b^ | 1.088 | 1 | .297 |  |  |
|  | Likelihood Ratio | 3.865 | 1 | .049 |  |  |
|  | Fisher's Exact Test |  |  |  | .298 | .149 |
|  | N of Valid Cases | 40 |  |  |  |  |
| 22-24.06 | Pearson Chi-Square | .017^e^ | 1 | .896 |  |  |
|  | Continuity Correction^b^ | .000 | 1 | 1.000 |  |  |
|  | Likelihood Ratio | .017 | 1 | .897 |  |  |
|  | Fisher's Exact Test |  |  |  | 1.000 | .668 |
|  | N of Valid Cases | 40 |  |  |  |  |
| 6-8.07 | Pearson Chi-Square | 3.265^f^ | 1 | .071 |  |  |
|  | Continuity Correction^b^ | 2.046 | 1 | .153 |  |  |
|  | Likelihood Ratio | 3.137 | 1 | .077 |  |  |
|  | Fisher's Exact Test |  |  |  | .130 | .078 |
|  | N of Valid Cases | 40 |  |  |  |  |
| 20-22.07 | Pearson Chi-Square | .077^g^ | 1 | .781 |  |  |
|  | Continuity Correction^b^ | .000 | 1 | 1.000 |  |  |
|  | Likelihood Ratio | .077 | 1 | .781 |  |  |
|  | Fisher's Exact Test |  |  |  | 1.000 | .529 |
|  | N of Valid Cases | 40 |  |  |  |  |
| 3-5.08 | Pearson Chi-Square | 2.533^h^ | 1 | .112 |  |  |
|  | Continuity Correction^b^ | 1.512 | 1 | .219 |  |  |
|  | Likelihood Ratio | 2.739 | 1 | .098 |  |  |
|  | Fisher's Exact Test |  |  |  | .157 | .108 |
|  | N of Valid Cases | 40 |  |  |  |  |
| 17-19.08 | Pearson Chi-Square | 3.832^f^ | 1 | .050 |  |  |
|  | Continuity Correction^b^ | 2.500 | 1 | .114 |  |  |
|  | Likelihood Ratio | 4.465 | 1 | .035 |  |  |
|  | Fisher's Exact Test |  |  |  | .067 | .052 |
|  | N of Valid Cases | 40 |  |  |  |  |
| 31.08-2.09 | Pearson Chi-Square | .119^i^ | 1 | .730 |  |  |
|  | Continuity Correction^b^ | .000 | 1 | 1.000 |  |  |
|  | Likelihood Ratio | .122 | 1 | .727 |  |  |
|  | Fisher's Exact Test |  |  |  | 1.000 | .548 |
|  | N of Valid Cases | 40 |  |  |  |  |
| 14-16.09 | Pearson Chi-Square | .635^j^ | 1 | .426 |  |  |
|  | Continuity Correction^b^ | .159 | 1 | .690 |  |  |
|  | Likelihood Ratio | .670 | 1 | .413 |  |  |
|  | Fisher's Exact Test |  |  |  | .693 | .355 |
|  | N of Valid Cases | 40 |  |  |  |  |
| Total | Pearson Chi-Square | .598^k^ | 1 | .440 |  |  |
|  | Continuity Correction^b^ | .084 | 1 | .772 |  |  |
|  | Likelihood Ratio | .656 | 1 | .418 |  |  |
|  | Fisher's Exact Test |  |  |  | .648 | .405 |
|  | N of Valid Cases | 40 |  |  |  |  |
| a. 1 cells (25.0%) have expected count less than 5. The minimum expected count is 2.70. | | | | | | |
| b. Computed only for a 2x2 table | | | | | | |
| c. 1 cells (25.0%) have expected count less than 5. The minimum expected count is 4.50. | | | | | | |
| d. 2 cells (50.0%) have expected count less than 5. The minimum expected count is 1.50. | | | | | | |
| e. 2 cells (50.0%) have expected count less than 5. The minimum expected count is .90. | | | | | | |
| f. 1 cells (25.0%) have expected count less than 5. The minimum expected count is 3.60. | | | | | | |
| g. 0 cells (0.0%) have expected count less than 5. The minimum expected count is 5.40. | | | | | | |
| h. 1 cells (25.0%) have expected count less than 5. The minimum expected count is 4.20. | | | | | | |
| i. 1 cells (25.0%) have expected count less than 5. The minimum expected count is 2.40. | | | | | | |
| j. 1 cells (25.0%) have expected count less than 5. The minimum expected count is 3.00. | | | | | | |
| k. 2 cells (50.0%) have expected count less than 5. The minimum expected count is 1.80. | | | | | | |

## Mean Abundance

| **Independent-Samples Mann-Whitney U Test Summary** | | |
| --- | --- | --- |
| 28-30.09 | Total N | 40 |
|  | Mann-Whitney U | 188.000 |
|  | Wilcoxon W | 594.000 |
|  | Test Statistic | 188.000 |
|  | Standard Error | 33.691 |
|  | Standardized Test Statistic | .594 |
|  | Asymptotic Sig.(2-sided test) | .553 |
|  | Exact Sig.(2-sided test) | .570 |
| 12-14.10 | Total N | 40 |
|  | Mann-Whitney U | 230.500 |
|  | Wilcoxon W | 636.500 |
|  | Test Statistic | 230.500 |
|  | Standard Error | 29.460 |
|  | Standardized Test Statistic | 2.122 |
|  | Asymptotic Sig.(2-sided test) | .034 |
|  | Exact Sig.(2-sided test) | .065 |
| 26-28.10 | Total N | 40 |
|  | Mann-Whitney U | 198.000 |
|  | Wilcoxon W | 604.000 |
|  | Test Statistic | 198.000 |
|  | Standard Error | 19.470 |
|  | Standardized Test Statistic | 1.541 |
|  | Asymptotic Sig.(2-sided test) | .123 |
|  | Exact Sig.(2-sided test) | .389 |
| 22-24.06 | Total N | 40 |
|  | Mann-Whitney U | 166.000 |
|  | Wilcoxon W | 572.000 |
|  | Test Statistic | 166.000 |
|  | Standard Error | 15.476 |
|  | Standardized Test Statistic | -.129 |
|  | Asymptotic Sig.(2-sided test) | .897 |
|  | Exact Sig.(2-sided test) | .965 |
| 6-8.07 | Total N | 40 |
|  | Mann-Whitney U | 113.000 |
|  | Wilcoxon W | 519.000 |
|  | Test Statistic | 113.000 |
|  | Standard Error | 27.466 |
|  | Standardized Test Statistic | -2.002 |
|  | Asymptotic Sig.(2-sided test) | .045 |
|  | Exact Sig.(2-sided test) | .108 |
| 20-22.07 | Total N | 40 |
|  | Mann-Whitney U | 189.000 |
|  | Wilcoxon W | 595.000 |
|  | Test Statistic | 189.000 |
|  | Standard Error | 32.304 |
|  | Standardized Test Statistic | .650 |
|  | Asymptotic Sig.(2-sided test) | .516 |
|  | Exact Sig.(2-sided test) | .550 |
| 3-5.08 | Total N | 40 |
|  | Mann-Whitney U | 169.500 |
|  | Wilcoxon W | 575.500 |
|  | Test Statistic | 169.500 |
|  | Standard Error | 33.150 |
|  | Standardized Test Statistic | .045 |
|  | Asymptotic Sig.(2-sided test) | .964 |
|  | Exact Sig.(2-sided test) | .965 |
| 17-19.08 | Total N | 40 |
|  | Mann-Whitney U | 158.500 |
|  | Wilcoxon W | 564.500 |
|  | Test Statistic | 158.500 |
|  | Standard Error | 33.423 |
|  | Standardized Test Statistic | -.284 |
|  | Asymptotic Sig.(2-sided test) | .776 |
|  | Exact Sig.(2-sided test) | .782 |
| 31.08-2.09 | Total N | 40 |
|  | Mann-Whitney U | 200.000 |
|  | Wilcoxon W | 606.000 |
|  | Test Statistic | 200.000 |
|  | Standard Error | 33.748 |
|  | Standardized Test Statistic | .948 |
|  | Asymptotic Sig.(2-sided test) | .343 |
|  | Exact Sig.(2-sided test) | .358 |
| 14-16.09 | Total N | 40 |
|  | Mann-Whitney U | 196.500 |
|  | Wilcoxon W | 602.500 |
|  | Test Statistic | 196.500 |
|  | Standard Error | 33.617 |
|  | Standardized Test Statistic | .848 |
|  | Asymptotic Sig.(2-sided test) | .397 |
|  | Exact Sig.(2-sided test) | .405 |
| Total | Total N | 40 |
|  | Mann-Whitney U | 205.500 |
|  | Wilcoxon W | 611.500 |
|  | Test Statistic | 205.500 |
|  | Standard Error | 33.826 |
|  | Standardized Test Statistic | 1.109 |
|  | Asymptotic Sig.(2-sided test) | .268 |
|  | Exact Sig.(2-sided test) | .273 |

## Mean intensity - significant

| **Hypothesis Test Summary** | | | | | | |
| --- | --- | --- | --- | --- | --- | --- |
| Date | Positive | | Null Hypothesis | Test | Sig.^a,b^ | Decision |
| 28-30.09 | 0 | 1 | The distribution of Total is the same across categories of Colour. | Independent-Samples Mann-Whitney U Test | 1.000^c^ | Retain the null hypothesis. |
|  | 1 | 1 | The distribution of Total is the same across categories of Colour. | Independent-Samples Mann-Whitney U Test | .025^c^ | Reject the null hypothesis. |
| 12-14.10 | 0 | 1 | The distribution of Total is the same across categories of Colour. | Independent-Samples Mann-Whitney U Test | 1.000^c^ | Retain the null hypothesis. |
|  | 1 | 1 | The distribution of Total is the same across categories of Colour. | Independent-Samples Mann-Whitney U Test | .019^c^ | Reject the null hypothesis. |
| 26-28.10 | 0 | 1 | The distribution of Total is the same across categories of Colour. | Independent-Samples Mann-Whitney U Test | 1.000^c^ | Retain the null hypothesis. |
| 22-24.06 | 0 | 1 | The distribution of Total is the same across categories of Colour. | Independent-Samples Mann-Whitney U Test | 1.000^c^ | Retain the null hypothesis. |
|  | 1 | 1 | The distribution of Total is the same across categories of Colour. | Independent-Samples Mann-Whitney U Test | 1.000^c^ | Retain the null hypothesis. |
| 6-8.07 | 0 | 1 | The distribution of Total is the same across categories of Colour. | Independent-Samples Mann-Whitney U Test | 1.000^c^ | Retain the null hypothesis. |
|  | 1 | 1 | The distribution of Total is the same across categories of Colour. | Independent-Samples Mann-Whitney U Test | .310^c^ | Retain the null hypothesis. |
| 20-22.07 | 0 | 1 | The distribution of Total is the same across categories of Colour. | Independent-Samples Mann-Whitney U Test | 1.000^c^ | Retain the null hypothesis. |
|  | 1 | 1 | The distribution of Total is the same across categories of Colour. | Independent-Samples Mann-Whitney U Test | .039^c^ | Reject the null hypothesis. |
| 3-5.08 | 0 | 1 | The distribution of Total is the same across categories of Colour. | Independent-Samples Mann-Whitney U Test | 1.000^c^ | Retain the null hypothesis. |
|  | 1 | 1 | The distribution of Total is the same across categories of Colour. | Independent-Samples Mann-Whitney U Test | .014^c^ | Reject the null hypothesis. |
| 17-19.08 | 0 | 1 | The distribution of Total is the same across categories of Colour. | Independent-Samples Mann-Whitney U Test | 1.000^c^ | Retain the null hypothesis. |
|  | 1 | 1 | The distribution of Total is the same across categories of Colour. | Independent-Samples Mann-Whitney U Test | .047^c^ | Reject the null hypothesis. |
| 31.08-2.09 | 0 | 1 | The distribution of Total is the same across categories of Colour. | Independent-Samples Mann-Whitney U Test | 1.000^c^ | Retain the null hypothesis. |
|  | 1 | 1 | The distribution of Total is the same across categories of Colour. | Independent-Samples Mann-Whitney U Test | .109^c^ | Retain the null hypothesis. |
| 14-16.09 | 0 | 1 | The distribution of Total is the same across categories of Colour. | Independent-Samples Mann-Whitney U Test | 1.000^c^ | Retain the null hypothesis. |
|  | 1 | 1 | The distribution of Total is the same across categories of Colour. | Independent-Samples Mann-Whitney U Test | .031^c^ | Reject the null hypothesis. |
| Total | 0 | 1 | The distribution of Total is the same across categories of Colour. | Independent-Samples Mann-Whitney U Test | 1.000^c^ | Retain the null hypothesis. |
|  | 1 | 1 | The distribution of Total is the same across categories of Colour. | Independent-Samples Mann-Whitney U Test | .050^c^ | Reject the null hypothesis. |
| a. The significance level is .050. | | | | | | |
| b. Asymptotic significance is displayed. | | | | | | |
| c. Exact significance is displayed for this test. | | | | | | |

**Independent-Samples Mann-Whitney U Test**

**Total across Colour**

| **Independent-Samples Mann-Whitney U Test Summary** | | | |
| --- | --- | --- | --- |
| 28-30.09 | 0 | Total N | 9 |
|  |  | Mann-Whitney U | 4.000 |
|  |  | Wilcoxon W | 40.000 |
|  |  | Test Statistic | 4.000 |
|  |  | Standard Error | .000 |
|  |  | Standardized Test Statistic | .000 |
|  |  | Asymptotic Sig.(2-sided test) | 1.000 |
|  |  | Exact Sig.(2-sided test) | 1.000 |
|  | 1 | Total N | 31 |
|  |  | Mann-Whitney U | 164.000 |
|  |  | Wilcoxon W | 374.000 |
|  |  | Test Statistic | 164.000 |
|  |  | Standard Error | 24.221 |
|  |  | Standardized Test Statistic | 2.229 |
|  |  | Asymptotic Sig.(2-sided test) | .026 |
|  |  | Exact Sig.(2-sided test) | .025 |
| 12-14.10 | 0 | Total N | 25 |
|  |  | Mann-Whitney U | 75.000 |
|  |  | Wilcoxon W | 195.000 |
|  |  | Test Statistic | 75.000 |
|  |  | Standard Error | .000 |
|  |  | Standardized Test Statistic | .000 |
|  |  | Asymptotic Sig.(2-sided test) | 1.000 |
|  |  | Exact Sig.(2-sided test) | 1.000 |
|  | 1 | Total N | 15 |
|  |  | Mann-Whitney U | 25.500 |
|  |  | Wilcoxon W | 116.500 |
|  |  | Test Statistic | 25.500 |
|  |  | Standard Error | 5.883 |
|  |  | Standardized Test Statistic | 2.125 |
|  |  | Asymptotic Sig.(2-sided test) | .034 |
|  |  | Exact Sig.(2-sided test) | .019 |
| 26-28.10 | 0 | Total N | 35 |
|  |  | Mann-Whitney U | 138.000 |
|  |  | Wilcoxon W | 414.000 |
|  |  | Test Statistic | 138.000 |
|  |  | Standard Error | .000 |
|  |  | Standardized Test Statistic | .000 |
|  |  | Asymptotic Sig.(2-sided test) | 1.000 |
|  |  | Exact Sig.(2-sided test) | 1.000 |
| 22-24.06 | 0 | Total N | 37 |
|  |  | Mann-Whitney U | 143.000 |
|  |  | Wilcoxon W | 494.000 |
|  |  | Test Statistic | 143.000 |
|  |  | Standard Error | .000 |
|  |  | Standardized Test Statistic | .000 |
|  |  | Asymptotic Sig.(2-sided test) | 1.000 |
|  |  | Exact Sig.(2-sided test) | 1.000 |
|  | 1 | Total N | 3 |
|  |  | Mann-Whitney U | 1.000 |
|  |  | Wilcoxon W | 4.000 |
|  |  | Test Statistic | 1.000 |
|  |  | Standard Error | .816 |
|  |  | Standardized Test Statistic | .000 |
|  |  | Asymptotic Sig.(2-sided test) | 1.000 |
|  |  | Exact Sig.(2-sided test) | 1.000 |
| 6-8.07 | 0 | Total N | 28 |
|  |  | Mann-Whitney U | 66.000 |
|  |  | Wilcoxon W | 319.000 |
|  |  | Test Statistic | 66.000 |
|  |  | Standard Error | .000 |
|  |  | Standardized Test Statistic | .000 |
|  |  | Asymptotic Sig.(2-sided test) | 1.000 |
|  |  | Exact Sig.(2-sided test) | 1.000 |
|  | 1 | Total N | 12 |
|  |  | Mann-Whitney U | 11.000 |
|  |  | Wilcoxon W | 32.000 |
|  |  | Test Statistic | 11.000 |
|  |  | Standard Error | 6.234 |
|  |  | Standardized Test Statistic | -1.123 |
|  |  | Asymptotic Sig.(2-sided test) | .261 |
|  |  | Exact Sig.(2-sided test) | .310 |
| 20-22.07 | 0 | Total N | 18 |
|  |  | Mann-Whitney U | 32.500 |
|  |  | Wilcoxon W | 123.500 |
|  |  | Test Statistic | 32.500 |
|  |  | Standard Error | .000 |
|  |  | Standardized Test Statistic | .000 |
|  |  | Asymptotic Sig.(2-sided test) | 1.000 |
|  |  | Exact Sig.(2-sided test) | 1.000 |
|  | 1 | Total N | 22 |
|  |  | Mann-Whitney U | 81.500 |
|  |  | Wilcoxon W | 201.500 |
|  |  | Test Statistic | 81.500 |
|  |  | Standard Error | 14.182 |
|  |  | Standardized Test Statistic | 2.045 |
|  |  | Asymptotic Sig.(2-sided test) | .041 |
|  |  | Exact Sig.(2-sided test) | .039 |
| 3-5.08 | 0 | Total N | 14 |
|  |  | Mann-Whitney U | 12.000 |
|  |  | Wilcoxon W | 90.000 |
|  |  | Test Statistic | 12.000 |
|  |  | Standard Error | .000 |
|  |  | Standardized Test Statistic | .000 |
|  |  | Asymptotic Sig.(2-sided test) | 1.000 |
|  |  | Exact Sig.(2-sided test) | 1.000 |
|  | 1 | Total N | 26 |
|  |  | Mann-Whitney U | 125.500 |
|  |  | Wilcoxon W | 261.500 |
|  |  | Test Statistic | 125.500 |
|  |  | Standard Error | 18.970 |
|  |  | Standardized Test Statistic | 2.398 |
|  |  | Asymptotic Sig.(2-sided test) | .016 |
|  |  | Exact Sig.(2-sided test) | .014 |
| 17-19.08 | 0 | Total N | 12 |
|  |  | Mann-Whitney U | 5.500 |
|  |  | Wilcoxon W | 71.500 |
|  |  | Test Statistic | 5.500 |
|  |  | Standard Error | .000 |
|  |  | Standardized Test Statistic | .000 |
|  |  | Asymptotic Sig.(2-sided test) | 1.000 |
|  |  | Exact Sig.(2-sided test) | 1.000 |
|  | 1 | Total N | 28 |
|  |  | Mann-Whitney U | 136.000 |
|  |  | Wilcoxon W | 289.000 |
|  |  | Test Statistic | 136.000 |
|  |  | Standard Error | 21.255 |
|  |  | Standardized Test Statistic | 1.999 |
|  |  | Asymptotic Sig.(2-sided test) | .046 |
|  |  | Exact Sig.(2-sided test) | .047 |
| 31.08-2.09 | 0 | Total N | 8 |
|  |  | Mann-Whitney U | 6.000 |
|  |  | Wilcoxon W | 27.000 |
|  |  | Test Statistic | 6.000 |
|  |  | Standard Error | .000 |
|  |  | Standardized Test Statistic | .000 |
|  |  | Asymptotic Sig.(2-sided test) | 1.000 |
|  |  | Exact Sig.(2-sided test) | 1.000 |
|  | 1 | Total N | 32 |
|  |  | Mann-Whitney U | 150.000 |
|  |  | Wilcoxon W | 403.000 |
|  |  | Test Statistic | 150.000 |
|  |  | Standard Error | 24.597 |
|  |  | Standardized Test Statistic | 1.626 |
|  |  | Asymptotic Sig.(2-sided test) | .104 |
|  |  | Exact Sig.(2-sided test) | .109 |
| 14-16.09 | 0 | Total N | 10 |
|  |  | Mann-Whitney U | 8.000 |
|  |  | Wilcoxon W | 44.000 |
|  |  | Test Statistic | 8.000 |
|  |  | Standard Error | .000 |
|  |  | Standardized Test Statistic | .000 |
|  |  | Asymptotic Sig.(2-sided test) | 1.000 |
|  |  | Exact Sig.(2-sided test) | 1.000 |
|  | 1 | Total N | 30 |
|  |  | Mann-Whitney U | 148.500 |
|  |  | Wilcoxon W | 358.500 |
|  |  | Test Statistic | 148.500 |
|  |  | Standard Error | 22.728 |
|  |  | Standardized Test Statistic | 2.134 |
|  |  | Asymptotic Sig.(2-sided test) | .033 |
|  |  | Exact Sig.(2-sided test) | .031 |
| Total | 0 | Total N | 6 |
|  |  | Mann-Whitney U | 2.500 |
|  |  | Wilcoxon W | 17.500 |
|  |  | Test Statistic | 2.500 |
|  |  | Standard Error | .000 |
|  |  | Standardized Test Statistic | .000 |
|  |  | Asymptotic Sig.(2-sided test) | 1.000 |
|  |  | Exact Sig.(2-sided test) | 1.000 |
|  | 1 | Total N | 34 |
|  |  | Mann-Whitney U | 180.000 |
|  |  | Wilcoxon W | 456.000 |
|  |  | Test Statistic | 180.000 |
|  |  | Standard Error | 27.165 |
|  |  | Standardized Test Statistic | 1.969 |
|  |  | Asymptotic Sig.(2-sided test) | .049 |
|  |  | Exact Sig.(2-sided test) | .050 |

# Husbandry

## Prevalence – significant

| **Case Processing Summary** |
| --- |

| **Crosstab** | | | | | |
| --- | --- | --- | --- | --- | --- |
| Count | | | | | |
| Date | | | Outdoor/Indoor | | Total |
|  |  |  | Indoor | Outodoor |  |
| 28-30.09 | Positive | 0 | 6 | 3 | 9 |
|  |  | 1 | 1 | 30 | 31 |
|  | Total | | 7 | 33 | 40 |
| 12-14.10 | Positive | 0 | 7 | 18 | 25 |
|  |  | 1 | 0 | 15 | 15 |
|  | Total | | 7 | 33 | 40 |
| 26-28.10 | Positive | 0 | 7 | 28 | 35 |
|  |  | 1 | 0 | 5 | 5 |
|  | Total | | 7 | 33 | 40 |
| 22-24.06 | Positive | 0 | 6 | 31 | 37 |
|  |  | 1 | 1 | 2 | 3 |
|  | Total | | 7 | 33 | 40 |
| 6-8.07 | Positive | 0 | 6 | 22 | 28 |
|  |  | 1 | 1 | 11 | 12 |
|  | Total | | 7 | 33 | 40 |
| 20-22.07 | Positive | 0 | 7 | 11 | 18 |
|  |  | 1 | 0 | 22 | 22 |
|  | Total | | 7 | 33 | 40 |
| 3-5.08 | Positive | 0 | 7 | 7 | 14 |
|  |  | 1 | 0 | 26 | 26 |
|  | Total | | 7 | 33 | 40 |
| 17-19.08 | Positive | 0 | 6 | 6 | 12 |
|  |  | 1 | 1 | 27 | 28 |
|  | Total | | 7 | 33 | 40 |
| 31.08-2.09 | Positive | 0 | 6 | 2 | 8 |
|  |  | 1 | 1 | 31 | 32 |
|  | Total | | 7 | 33 | 40 |
| 14-16.09 | Positive | 0 | 5 | 5 | 10 |
|  |  | 1 | 2 | 28 | 30 |
|  | Total | | 7 | 33 | 40 |
| Total | Positive | 0 | 5 | 1 | 6 |
|  |  | 1 | 2 | 32 | 34 |
|  | Total | | 7 | 33 | 40 |

| **Chi-Square Tests** | | | | | | |
| --- | --- | --- | --- | --- | --- | --- |
| Date | | Value | df | Asymptotic Significance (2-sided) | Exact Sig. (2-sided) | Exact Sig. (1-sided) |
| 28-30.09 | Pearson Chi-Square | 19.444^a^ | 1 | .000 |  |  |
|  | Continuity Correction^b^ | 15.298 | 1 | .000 |  |  |
|  | Likelihood Ratio | 16.805 | 1 | .000 |  |  |
|  | Fisher's Exact Test |  |  |  | .000 | .000 |
|  | N of Valid Cases | 40 |  |  |  |  |
| 12-14.10 | Pearson Chi-Square | 5.091^c^ | 1 | .024 |  |  |
|  | Continuity Correction^b^ | 3.336 | 1 | .068 |  |  |
|  | Likelihood Ratio | 7.450 | 1 | .006 |  |  |
|  | Fisher's Exact Test |  |  |  | .033 | .026 |
|  | N of Valid Cases | 40 |  |  |  |  |
| 26-28.10 | Pearson Chi-Square | 1.212^d^ | 1 | .271 |  |  |
|  | Continuity Correction^b^ | .223 | 1 | .637 |  |  |
|  | Likelihood Ratio | 2.070 | 1 | .150 |  |  |
|  | Fisher's Exact Test |  |  |  | .565 | .361 |
|  | N of Valid Cases | 40 |  |  |  |  |
| 22-24.06 | Pearson Chi-Square | .563^e^ | 1 | .453 |  |  |
|  | Continuity Correction^b^ | .000 | 1 | 1.000 |  |  |
|  | Likelihood Ratio | .479 | 1 | .489 |  |  |
|  | Fisher's Exact Test |  |  |  | .448 | .448 |
|  | N of Valid Cases | 40 |  |  |  |  |
| 6-8.07 | Pearson Chi-Square | .998^f^ | 1 | .318 |  |  |
|  | Continuity Correction^b^ | .297 | 1 | .586 |  |  |
|  | Likelihood Ratio | 1.118 | 1 | .290 |  |  |
|  | Fisher's Exact Test |  |  |  | .652 | .306 |
|  | N of Valid Cases | 40 |  |  |  |  |
| 20-22.07 | Pearson Chi-Square | 10.370^g^ | 1 | .001 |  |  |
|  | Continuity Correction^b^ | 7.852 | 1 | .005 |  |  |
|  | Likelihood Ratio | 13.041 | 1 | .000 |  |  |
|  | Fisher's Exact Test |  |  |  | .002 | .002 |
|  | N of Valid Cases | 40 |  |  |  |  |
| 3-5.08 | Pearson Chi-Square | 15.758^h^ | 1 | .000 |  |  |
|  | Continuity Correction^b^ | 12.485 | 1 | .000 |  |  |
|  | Likelihood Ratio | 17.690 | 1 | .000 |  |  |
|  | Fisher's Exact Test |  |  |  | .000 | .000 |
|  | N of Valid Cases | 40 |  |  |  |  |
| 17-19.08 | Pearson Chi-Square | 12.542^f^ | 1 | .000 |  |  |
|  | Continuity Correction^b^ | 9.532 | 1 | .002 |  |  |
|  | Likelihood Ratio | 11.834 | 1 | .001 |  |  |
|  | Fisher's Exact Test |  |  |  | .001 | .001 |
|  | N of Valid Cases | 40 |  |  |  |  |
| 31.08-2.09 | Pearson Chi-Square | 22.900^i^ | 1 | .000 |  |  |
|  | Continuity Correction^b^ | 18.193 | 1 | .000 |  |  |
|  | Likelihood Ratio | 19.201 | 1 | .000 |  |  |
|  | Fisher's Exact Test |  |  |  | .000 | .000 |
|  | N of Valid Cases | 40 |  |  |  |  |
| 14-16.09 | Pearson Chi-Square | 9.755^j^ | 1 | .002 |  |  |
|  | Continuity Correction^b^ | 6.984 | 1 | .008 |  |  |
|  | Likelihood Ratio | 8.539 | 1 | .003 |  |  |
|  | Fisher's Exact Test |  |  |  | .006 | .006 |
|  | N of Valid Cases | 40 |  |  |  |  |
| Total | Pearson Chi-Square | 21.190^k^ | 1 | .000 |  |  |
|  | Continuity Correction^b^ | 16.165 | 1 | .000 |  |  |
|  | Likelihood Ratio | 16.479 | 1 | .000 |  |  |
|  | Fisher's Exact Test |  |  |  | .000 | .000 |
|  | N of Valid Cases | 40 |  |  |  |  |
| a. 1 cells (25.0%) have expected count less than 5. The minimum expected count is 1.58. | | | | | | |
| b. Computed only for a 2x2 table | | | | | | |
| c. 2 cells (50.0%) have expected count less than 5. The minimum expected count is 2.63. | | | | | | |
| d. 2 cells (50.0%) have expected count less than 5. The minimum expected count is .88. | | | | | | |
| e. 2 cells (50.0%) have expected count less than 5. The minimum expected count is .52. | | | | | | |
| f. 2 cells (50.0%) have expected count less than 5. The minimum expected count is 2.10. | | | | | | |
| g. 2 cells (50.0%) have expected count less than 5. The minimum expected count is 3.15. | | | | | | |
| h. 2 cells (50.0%) have expected count less than 5. The minimum expected count is 2.45. | | | | | | |
| i. 1 cells (25.0%) have expected count less than 5. The minimum expected count is 1.40. | | | | | | |
| j. 1 cells (25.0%) have expected count less than 5. The minimum expected count is 1.75. | | | | | | |
| k. 2 cells (50.0%) have expected count less than 5. The minimum expected count is 1.05. | | | | | | |

## Mean Abundance -significant

| **Independent-Samples Mann-Whitney U Test Summary** | | |
| --- | --- | --- |
| 28-30.09 | Total N | 40 |
|  | Mann-Whitney U | 219.000 |
|  | Wilcoxon W | 780.000 |
|  | Test Statistic | 219.000 |
|  | Standard Error | 27.935 |
|  | Standardized Test Statistic | 3.705 |
|  | Asymptotic Sig.(2-sided test) | .000 |
|  | Exact Sig.(2-sided test) | .000 |
| 12-14.10 | Total N | 40 |
|  | Mann-Whitney U | 168.000 |
|  | Wilcoxon W | 729.000 |
|  | Test Statistic | 168.000 |
|  | Standard Error | 24.427 |
|  | Standardized Test Statistic | 2.149 |
|  | Asymptotic Sig.(2-sided test) | .032 |
|  | Exact Sig.(2-sided test) | .063 |
| 26-28.10 | Total N | 40 |
|  | Mann-Whitney U | 133.000 |
|  | Wilcoxon W | 694.000 |
|  | Test Statistic | 133.000 |
|  | Standard Error | 16.144 |
|  | Standardized Test Statistic | 1.084 |
|  | Asymptotic Sig.(2-sided test) | .278 |
|  | Exact Sig.(2-sided test) | .553 |
| 22-24.06 | Total N | 40 |
|  | Mann-Whitney U | 107.000 |
|  | Wilcoxon W | 668.000 |
|  | Test Statistic | 107.000 |
|  | Standard Error | 12.832 |
|  | Standardized Test Statistic | -.662 |
|  | Asymptotic Sig.(2-sided test) | .508 |
|  | Exact Sig.(2-sided test) | .781 |
| 6-8.07 | Total N | 40 |
|  | Mann-Whitney U | 142.500 |
|  | Wilcoxon W | 703.500 |
|  | Test Statistic | 142.500 |
|  | Standard Error | 22.774 |
|  | Standardized Test Statistic | 1.186 |
|  | Asymptotic Sig.(2-sided test) | .236 |
|  | Exact Sig.(2-sided test) | .344 |
| 20-22.07 | Total N | 40 |
|  | Mann-Whitney U | 192.500 |
|  | Wilcoxon W | 753.500 |
|  | Test Statistic | 192.500 |
|  | Standard Error | 26.785 |
|  | Standardized Test Statistic | 2.875 |
|  | Asymptotic Sig.(2-sided test) | .004 |
|  | Exact Sig.(2-sided test) | .004 |
| 3-5.08 | Total N | 40 |
|  | Mann-Whitney U | 206.500 |
|  | Wilcoxon W | 767.500 |
|  | Test Statistic | 206.500 |
|  | Standard Error | 27.486 |
|  | Standardized Test Statistic | 3.311 |
|  | Asymptotic Sig.(2-sided test) | .001 |
|  | Exact Sig.(2-sided test) | .000 |
| 17-19.08 | Total N | 40 |
|  | Mann-Whitney U | 207.000 |
|  | Wilcoxon W | 768.000 |
|  | Test Statistic | 207.000 |
|  | Standard Error | 27.713 |
|  | Standardized Test Statistic | 3.302 |
|  | Asymptotic Sig.(2-sided test) | .001 |
|  | Exact Sig.(2-sided test) | .000 |
| 31.08-2.09 | Total N | 40 |
|  | Mann-Whitney U | 223.000 |
|  | Wilcoxon W | 784.000 |
|  | Test Statistic | 223.000 |
|  | Standard Error | 27.983 |
|  | Standardized Test Statistic | 3.842 |
|  | Asymptotic Sig.(2-sided test) | .000 |
|  | Exact Sig.(2-sided test) | .000 |
| 14-16.09 | Total N | 40 |
|  | Mann-Whitney U | 206.500 |
|  | Wilcoxon W | 767.500 |
|  | Test Statistic | 206.500 |
|  | Standard Error | 27.874 |
|  | Standardized Test Statistic | 3.265 |
|  | Asymptotic Sig.(2-sided test) | .001 |
|  | Exact Sig.(2-sided test) | .000 |
| Total | Total N | 40 |
|  | Mann-Whitney U | 226.500 |
|  | Wilcoxon W | 787.500 |
|  | Test Statistic | 226.500 |
|  | Standard Error | 28.047 |
|  | Standardized Test Statistic | 3.958 |
|  | Asymptotic Sig.(2-sided test) | .000 |
|  | Exact Sig.(2-sided test) | .000 |

## Mean intensity - significant

| **Hypothesis Test Summary** | | | | | | |
| --- | --- | --- | --- | --- | --- | --- |
| Date | Positive | | Null Hypothesis | Test | Sig.^a,b^ | Decision |
| 28-30.09 | 0 | 1 | The distribution of Total is the same across categories of Outdoor/Indoor. | Independent-Samples Mann-Whitney U Test | 1.000^c^ | Retain the null hypothesis. |
|  | 1 | 1 | The distribution of Total is the same across categories of Outdoor/Indoor. | Independent-Samples Mann-Whitney U Test | .065^c^ | Retain the null hypothesis. |
| 12-14.10 | 0 | 1 | The distribution of Total is the same across categories of Outdoor/Indoor. | Independent-Samples Mann-Whitney U Test | 1.000^c^ | Retain the null hypothesis. |
| 26-28.10 | 0 | 1 | The distribution of Total is the same across categories of Outdoor/Indoor. | Independent-Samples Mann-Whitney U Test | 1.000^c^ | Retain the null hypothesis. |
| 22-24.06 | 0 | 1 | The distribution of Total is the same across categories of Outdoor/Indoor. | Independent-Samples Mann-Whitney U Test | 1.000^c^ | Retain the null hypothesis. |
|  | 1 | 1 | The distribution of Total is the same across categories of Outdoor/Indoor. | Independent-Samples Mann-Whitney U Test | 1.000^c^ | Retain the null hypothesis. |
| 6-8.07 | 0 | 1 | The distribution of Total is the same across categories of Outdoor/Indoor. | Independent-Samples Mann-Whitney U Test | 1.000^c^ | Retain the null hypothesis. |
|  | 1 | 1 | The distribution of Total is the same across categories of Outdoor/Indoor. | Independent-Samples Mann-Whitney U Test | .167^c^ | Retain the null hypothesis. |
| 20-22.07 | 0 | 1 | The distribution of Total is the same across categories of Outdoor/Indoor. | Independent-Samples Mann-Whitney U Test | 1.000^c^ | Retain the null hypothesis. |
| 3-5.08 | 0 | 1 | The distribution of Total is the same across categories of Outdoor/Indoor. | Independent-Samples Mann-Whitney U Test | 1.000^c^ | Retain the null hypothesis. |
| 17-19.08 | 0 | 1 | The distribution of Total is the same across categories of Outdoor/Indoor. | Independent-Samples Mann-Whitney U Test | 1.000^c^ | Retain the null hypothesis. |
|  | 1 | 1 | The distribution of Total is the same across categories of Outdoor/Indoor. | Independent-Samples Mann-Whitney U Test | .071^c^ | Retain the null hypothesis. |
| 31.08-2.09 | 0 | 1 | The distribution of Total is the same across categories of Outdoor/Indoor. | Independent-Samples Mann-Whitney U Test | 1.000^c^ | Retain the null hypothesis. |
|  | 1 | 1 | The distribution of Total is the same across categories of Outdoor/Indoor. | Independent-Samples Mann-Whitney U Test | .063^c^ | Retain the null hypothesis. |
| 31.08-2.09 | 0 | 1 | The distribution of Total is the same across categories of Outdoor/Indoor. | Independent-Samples Mann-Whitney U Test | 1.000^c^ | Retain the null hypothesis. |
|  | 1 | 1 | The distribution of Total is the same across categories of Outdoor/Indoor. | Independent-Samples Mann-Whitney U Test | .018^c^ | Reject the null hypothesis. |
| Total | 0 | 1 | The distribution of Total is the same across categories of Outdoor/Indoor. | Independent-Samples Mann-Whitney U Test | 1.000^c^ | Retain the null hypothesis. |
|  | 1 | 1 | The distribution of Total is the same across categories of Outdoor/Indoor. | Independent-Samples Mann-Whitney U Test | .004^c^ | Reject the null hypothesis. |
| a. The significance level is .050. | | | | | | |
| b. Asymptotic significance is displayed. | | | | | | |
| c. Exact significance is displayed for this test. | | | | | | |

**Independent-Samples Mann-Whitney U Test**

**Total across Outdoor/Indoor**

| **Independent-Samples Mann-Whitney U Test Summary** | | | |
| --- | --- | --- | --- |
| 28-30.09 | 0 | Total N | 9 |
|  |  | Mann-Whitney U | 9.000 |
|  |  | Wilcoxon W | 15.000 |
|  |  | Test Statistic | 9.000 |
|  |  | Standard Error | .000 |
|  |  | Standardized Test Statistic | .000 |
|  |  | Asymptotic Sig.(2-sided test) | 1.000 |
|  |  | Exact Sig.(2-sided test) | 1.000 |
|  | 1 | Total N | 31 |
|  |  | Mann-Whitney U | 30.000 |
|  |  | Wilcoxon W | 495.000 |
|  |  | Test Statistic | 30.000 |
|  |  | Standard Error | 8.944 |
|  |  | Standardized Test Statistic | 1.677 |
|  |  | Asymptotic Sig.(2-sided test) | .094 |
|  |  | Exact Sig.(2-sided test) | .065 |
| 12-14.10 | 0 | Total N | 25 |
|  |  | Mann-Whitney U | 63.000 |
|  |  | Wilcoxon W | 234.000 |
|  |  | Test Statistic | 63.000 |
|  |  | Standard Error | .000 |
|  |  | Standardized Test Statistic | .000 |
|  |  | Asymptotic Sig.(2-sided test) | 1.000 |
|  |  | Exact Sig.(2-sided test) | 1.000 |
| 26-28.10 | 0 | Total N | 35 |
|  |  | Mann-Whitney U | 98.000 |
|  |  | Wilcoxon W | 504.000 |
|  |  | Test Statistic | 98.000 |
|  |  | Standard Error | .000 |
|  |  | Standardized Test Statistic | .000 |
|  |  | Asymptotic Sig.(2-sided test) | 1.000 |
|  |  | Exact Sig.(2-sided test) | 1.000 |
| 22-24.06 | 0 | Total N | 37 |
|  |  | Mann-Whitney U | 93.000 |
|  |  | Wilcoxon W | 589.000 |
|  |  | Test Statistic | 93.000 |
|  |  | Standard Error | .000 |
|  |  | Standardized Test Statistic | .000 |
|  |  | Asymptotic Sig.(2-sided test) | 1.000 |
|  |  | Exact Sig.(2-sided test) | 1.000 |
|  | 1 | Total N | 3 |
|  |  | Mann-Whitney U | 2.000 |
|  |  | Wilcoxon W | 5.000 |
|  |  | Test Statistic | 2.000 |
|  |  | Standard Error | .816 |
|  |  | Standardized Test Statistic | 1.225 |
|  |  | Asymptotic Sig.(2-sided test) | .221 |
|  |  | Exact Sig.(2-sided test) | 1.000 |
| 6-8.07 | 0 | Total N | 28 |
|  |  | Mann-Whitney U | 66.000 |
|  |  | Wilcoxon W | 319.000 |
|  |  | Test Statistic | 66.000 |
|  |  | Standard Error | .000 |
|  |  | Standardized Test Statistic | .000 |
|  |  | Asymptotic Sig.(2-sided test) | 1.000 |
|  |  | Exact Sig.(2-sided test) | 1.000 |
|  | 1 | Total N | 12 |
|  |  | Mann-Whitney U | 10.500 |
|  |  | Wilcoxon W | 76.500 |
|  |  | Test Statistic | 10.500 |
|  |  | Standard Error | 3.446 |
|  |  | Standardized Test Statistic | 1.451 |
|  |  | Asymptotic Sig.(2-sided test) | .147 |
|  |  | Exact Sig.(2-sided test) | .167 |
| 20-22.07 | 0 | Total N | 18 |
|  |  | Mann-Whitney U | 38.500 |
|  |  | Wilcoxon W | 104.500 |
|  |  | Test Statistic | 38.500 |
|  |  | Standard Error | .000 |
|  |  | Standardized Test Statistic | .000 |
|  |  | Asymptotic Sig.(2-sided test) | 1.000 |
|  |  | Exact Sig.(2-sided test) | 1.000 |
| 3-5.08 | 0 | Total N | 14 |
|  |  | Mann-Whitney U | 24.500 |
|  |  | Wilcoxon W | 52.500 |
|  |  | Test Statistic | 24.500 |
|  |  | Standard Error | .000 |
|  |  | Standardized Test Statistic | .000 |
|  |  | Asymptotic Sig.(2-sided test) | 1.000 |
|  |  | Exact Sig.(2-sided test) | 1.000 |
| 17-19.08 | 0 | Total N | 12 |
|  |  | Mann-Whitney U | 18.000 |
|  |  | Wilcoxon W | 39.000 |
|  |  | Test Statistic | 18.000 |
|  |  | Standard Error | .000 |
|  |  | Standardized Test Statistic | .000 |
|  |  | Asymptotic Sig.(2-sided test) | 1.000 |
|  |  | Exact Sig.(2-sided test) | 1.000 |
|  | 1 | Total N | 28 |
|  |  | Mann-Whitney U | 27.000 |
|  |  | Wilcoxon W | 405.000 |
|  |  | Test Statistic | 27.000 |
|  |  | Standard Error | 8.077 |
|  |  | Standardized Test Statistic | 1.671 |
|  |  | Asymptotic Sig.(2-sided test) | .095 |
|  |  | Exact Sig.(2-sided test) | .071 |
| 31.08-2.09 | 0 | Total N | 8 |
|  |  | Mann-Whitney U | 6.000 |
|  |  | Wilcoxon W | 9.000 |
|  |  | Test Statistic | 6.000 |
|  |  | Standard Error | .000 |
|  |  | Standardized Test Statistic | .000 |
|  |  | Asymptotic Sig.(2-sided test) | 1.000 |
|  |  | Exact Sig.(2-sided test) | 1.000 |
|  | 1 | Total N | 32 |
|  |  | Mann-Whitney U | 31.000 |
|  |  | Wilcoxon W | 527.000 |
|  |  | Test Statistic | 31.000 |
|  |  | Standard Error | 9.233 |
|  |  | Standardized Test Statistic | 1.679 |
|  |  | Asymptotic Sig.(2-sided test) | .093 |
|  |  | Exact Sig.(2-sided test) | .063 |
| 14-16.09 | 0 | Total N | 10 |
|  |  | Mann-Whitney U | 12.500 |
|  |  | Wilcoxon W | 27.500 |
|  |  | Test Statistic | 12.500 |
|  |  | Standard Error | .000 |
|  |  | Standardized Test Statistic | .000 |
|  |  | Asymptotic Sig.(2-sided test) | 1.000 |
|  |  | Exact Sig.(2-sided test) | 1.000 |
|  | 1 | Total N | 30 |
|  |  | Mann-Whitney U | 54.000 |
|  |  | Wilcoxon W | 460.000 |
|  |  | Test Statistic | 54.000 |
|  |  | Standard Error | 12.026 |
|  |  | Standardized Test Statistic | 2.162 |
|  |  | Asymptotic Sig.(2-sided test) | .031 |
|  |  | Exact Sig.(2-sided test) | .018 |
| Total | 0 | Total N | 6 |
|  |  | Mann-Whitney U | 2.500 |
|  |  | Wilcoxon W | 3.500 |
|  |  | Test Statistic | 2.500 |
|  |  | Standard Error | .000 |
|  |  | Standardized Test Statistic | .000 |
|  |  | Asymptotic Sig.(2-sided test) | 1.000 |
|  |  | Exact Sig.(2-sided test) | 1.000 |
|  | 1 | Total N | 34 |
|  |  | Mann-Whitney U | 64.000 |
|  |  | Wilcoxon W | 592.000 |
|  |  | Test Statistic | 64.000 |
|  |  | Standard Error | 13.663 |
|  |  | Standardized Test Statistic | 2.342 |
|  |  | Asymptotic Sig.(2-sided test) | .019 |
|  |  | Exact Sig.(2-sided test) | .004 |

# Anatomical Regions

## Mean intensity – anatomical regions – significant

| **Hypothesis Test Summary** | | | | | | |
| --- | --- | --- | --- | --- | --- | --- |
| Positive | Date | | Null Hypothesis | Test | Sig.^a,b^ | Decision |
| 1 | 28-30.09 | 1 | The distributions of Foreleg, Hindleg, Head+Neck, Thorax and Abdomen are the same. | Related-Samples Friedman's Two-Way Analysis of Variance by Ranks | .000 | Reject the null hypothesis. |
|  | 12-14.10 | 1 | The distributions of Foreleg, Hindleg, Head+Neck, Thorax and Abdomen are the same. | Related-Samples Friedman's Two-Way Analysis of Variance by Ranks | .000 | Reject the null hypothesis. |
|  | 26-28.10 | 1 | The distributions of Foreleg, Hindleg, Head+Neck, Thorax and Abdomen are the same. | Related-Samples Friedman's Two-Way Analysis of Variance by Ranks | .003 | Reject the null hypothesis. |
|  | 22-24.06 | 1 | The distributions of Foreleg, Hindleg, Head+Neck, Thorax and Abdomen are the same. | Related-Samples Friedman's Two-Way Analysis of Variance by Ranks | .017 | Reject the null hypothesis. |
|  | 6-8.07 | 1 | The distributions of Foreleg, Hindleg, Head+Neck, Thorax and Abdomen are the same. | Related-Samples Friedman's Two-Way Analysis of Variance by Ranks | .000 | Reject the null hypothesis. |
|  | 20-22.07 | 1 | The distributions of Foreleg, Hindleg, Head+Neck, Thorax and Abdomen are the same. | Related-Samples Friedman's Two-Way Analysis of Variance by Ranks | .000 | Reject the null hypothesis. |
|  | 3-5.08 | 1 | The distributions of Foreleg, Hindleg, Head+Neck, Thorax and Abdomen are the same. | Related-Samples Friedman's Two-Way Analysis of Variance by Ranks | .000 | Reject the null hypothesis. |
|  | 17-19.08 | 1 | The distributions of Foreleg, Hindleg, Head+Neck, Thorax and Abdomen are the same. | Related-Samples Friedman's Two-Way Analysis of Variance by Ranks | .000 | Reject the null hypothesis. |
|  | 31.08-2.09 | 1 | The distributions of Foreleg, Hindleg, Head+Neck, Thorax and Abdomen are the same. | Related-Samples Friedman's Two-Way Analysis of Variance by Ranks | .000 | Reject the null hypothesis. |
|  | 14-16.09 | 1 | The distributions of Foreleg, Hindleg, Head+Neck, Thorax and Abdomen are the same. | Related-Samples Friedman's Two-Way Analysis of Variance by Ranks | .000 | Reject the null hypothesis. |
|  | Total | 1 | The distributions of Foreleg, Hindleg, Head+Neck, Thorax and Abdomen are the same. | Related-Samples Friedman's Two-Way Analysis of Variance by Ranks | .000 | Reject the null hypothesis. |
| a. The significance level is .050. | | | | | | |
| b. Asymptotic significance is displayed. | | | | | | |

| **Related-Samples Friedman's Two-Way Analysis of Variance by Ranks Summary** | | | |
| --- | --- | --- | --- |
| 1 | 28-30.09 | Total N | 31 |
|  |  | Test Statistic | 92.144 |
|  |  | Degree Of Freedom | 4 |
|  |  | Asymptotic Sig.(2-sided test) | .000 |
|  | 12-14.10 | Total N | 15 |
|  |  | Test Statistic | 28.813 |
|  |  | Degree Of Freedom | 4 |
|  |  | Asymptotic Sig.(2-sided test) | .000 |
|  | 26-28.10 | Total N | 5 |
|  |  | Test Statistic | 16.260 |
|  |  | Degree Of Freedom | 4 |
|  |  | Asymptotic Sig.(2-sided test) | .003 |
|  | 22-24.06 | Total N | 3 |
|  |  | Test Statistic | 12.000 |
|  |  | Degree Of Freedom | 4 |
|  |  | Asymptotic Sig.(2-sided test) | .017 |
|  | 6-8.07 | Total N | 12 |
|  |  | Test Statistic | 30.984 |
|  |  | Degree Of Freedom | 4 |
|  |  | Asymptotic Sig.(2-sided test) | .000 |
|  | 20-22.07 | Total N | 22 |
|  |  | Test Statistic | 79.111 |
|  |  | Degree Of Freedom | 4 |
|  |  | Asymptotic Sig.(2-sided test) | .000 |
|  | 3-5.08 | Total N | 26 |
|  |  | Test Statistic | 83.381 |
|  |  | Degree Of Freedom | 4 |
|  |  | Asymptotic Sig.(2-sided test) | .000 |
|  | 17-19.08 | Total N | 28 |
|  |  | Test Statistic | 89.900 |
|  |  | Degree Of Freedom | 4 |
|  |  | Asymptotic Sig.(2-sided test) | .000 |
|  | 31.08-2.09 | Total N | 32 |
|  |  | Test Statistic | 86.959 |
|  |  | Degree Of Freedom | 4 |
|  |  | Asymptotic Sig.(2-sided test) | .000 |
|  | 14-16.09 | Total N | 30 |
|  |  | Test Statistic | 91.557 |
|  |  | Degree Of Freedom | 4 |
|  |  | Asymptotic Sig.(2-sided test) | .000 |
|  | Total | Total N | 34 |
|  |  | Test Statistic | 102.700 |
|  |  | Degree Of Freedom | 4 |
|  |  | Asymptotic Sig.(2-sided test) | .000 |
| a. Multiple comparisons are not performed because the overall test retained the null hypothesis of no differences. | | | |

| **Pairwise Comparisons** | | | | | | | |
| --- | --- | --- | --- | --- | --- | --- | --- |
| Positive | Date | Sample 1-Sample 2 | Test Statistic | Std. Error | Std. Test Statistic | Sig. | Adj. Sig.^a^ |
| 1 | 28-30.09 | Hindleg-Head+Neck | -.306 | .402 | -.763 | .445 | 1.000 |
|  |  | Hindleg-Abdomen | -.839 | .402 | -2.088 | .037 | .368 |
|  |  | Hindleg-Thorax | -1.500 | .402 | -3.735 | .000 | .002 |
|  |  | Hindleg-Foreleg | 3.081 | .402 | 7.671 | .000 | .000 |
|  |  | Head+Neck-Abdomen | -.532 | .402 | -1.325 | .185 | 1.000 |
|  |  | Head+Neck-Thorax | -1.194 | .402 | -2.972 | .003 | .030 |
|  |  | Head+Neck-Foreleg | 2.774 | .402 | 6.908 | .000 | .000 |
|  |  | Abdomen-Thorax | .661 | .402 | 1.647 | .100 | .996 |
|  |  | Abdomen-Foreleg | 2.242 | .402 | 5.582 | .000 | .000 |
|  |  | Thorax-Foreleg | 1.581 | .402 | 3.936 | .000 | .001 |
|  | 12-14.10 | Hindleg-Head+Neck | -.067 | .577 | -.115 | .908 | 1.000 |
|  |  | Hindleg-Abdomen | -1.467 | .577 | -2.540 | .011 | .111 |
|  |  | Hindleg-Thorax | -2.133 | .577 | -3.695 | .000 | .002 |
|  |  | Hindleg-Foreleg | 1.833 | .577 | 3.175 | .001 | .015 |
|  |  | Head+Neck-Abdomen | -1.400 | .577 | -2.425 | .015 | .153 |
|  |  | Head+Neck-Thorax | -2.067 | .577 | -3.580 | .000 | .003 |
|  |  | Head+Neck-Foreleg | 1.767 | .577 | 3.060 | .002 | .022 |
|  |  | Abdomen-Thorax | .667 | .577 | 1.155 | .248 | 1.000 |
|  |  | Abdomen-Foreleg | .367 | .577 | .635 | .525 | 1.000 |
|  |  | Foreleg-Thorax | -.300 | .577 | -.520 | .603 | 1.000 |
|  | 26-28.10 | Hindleg-Abdomen | -1.800 | 1.000 | -1.800 | .072 | .719 |
|  |  | Hindleg-Thorax | -2.300 | 1.000 | -2.300 | .021 | .214 |
|  |  | Head+Neck-Abdomen | -2.100 | 1.000 | -2.100 | .036 | .357 |
|  |  | Head+Neck-Thorax | -2.600 | 1.000 | -2.600 | .009 | .093 |
|  |  | Abdomen-Thorax | .500 | 1.000 | .500 | .617 | 1.000 |
|  |  | Foreleg-Thorax | -2.600 | 1.000 | -2.600 | .009 | .093 |
|  |  | Foreleg-Head+Neck | .000 | 1.000 | .000 | 1.000 | 1.000 |
|  |  | Foreleg-Hindleg | -.300 | 1.000 | -.300 | .764 | 1.000 |
|  |  | Foreleg-Abdomen | -2.100 | 1.000 | -2.100 | .036 | .357 |
|  |  | Head+Neck-Hindleg | .300 | 1.000 | .300 | .764 | 1.000 |
|  | 22-24.06 | Hindleg-Head+Neck | .000 | 1.291 | .000 | 1.000 | 1.000 |
|  |  | Hindleg-Abdomen | .000 | 1.291 | .000 | 1.000 | 1.000 |
|  |  | Hindleg-Thorax | .000 | 1.291 | .000 | 1.000 | 1.000 |
|  |  | Hindleg-Foreleg | 2.500 | 1.291 | 1.936 | .053 | .528 |
|  |  | Head+Neck-Abdomen | .000 | 1.291 | .000 | 1.000 | 1.000 |
|  |  | Head+Neck-Thorax | .000 | 1.291 | .000 | 1.000 | 1.000 |
|  |  | Head+Neck-Foreleg | 2.500 | 1.291 | 1.936 | .053 | .528 |
|  |  | Abdomen-Foreleg | 2.500 | 1.291 | 1.936 | .053 | .528 |
|  |  | Thorax-Foreleg | 2.500 | 1.291 | 1.936 | .053 | .528 |
|  |  | Thorax-Abdomen | .000 | 1.291 | .000 | 1.000 | 1.000 |
|  | 6-8.07 | Hindleg-Head+Neck | .000 | .645 | .000 | 1.000 | 1.000 |
|  |  | Hindleg-Abdomen | .000 | .645 | .000 | 1.000 | 1.000 |
|  |  | Hindleg-Thorax | -.583 | .645 | -.904 | .366 | 1.000 |
|  |  | Hindleg-Foreleg | 2.125 | .645 | 3.292 | .001 | .010 |
|  |  | Head+Neck-Abdomen | .000 | .645 | .000 | 1.000 | 1.000 |
|  |  | Head+Neck-Thorax | -.583 | .645 | -.904 | .366 | 1.000 |
|  |  | Head+Neck-Foreleg | 2.125 | .645 | 3.292 | .001 | .010 |
|  |  | Abdomen-Thorax | .583 | .645 | .904 | .366 | 1.000 |
|  |  | Abdomen-Foreleg | 2.125 | .645 | 3.292 | .001 | .010 |
|  |  | Thorax-Foreleg | 1.542 | .645 | 2.388 | .017 | .169 |
|  | 20-22.07 | Hindleg-Head+Neck | -.136 | .477 | -.286 | .775 | 1.000 |
|  |  | Hindleg-Abdomen | .000 | .477 | .000 | 1.000 | 1.000 |
|  |  | Hindleg-Thorax | -.341 | .477 | -.715 | .475 | 1.000 |
|  |  | Hindleg-Foreleg | 2.591 | .477 | 5.435 | .000 | .000 |
|  |  | Head+Neck-Thorax | -.205 | .477 | -.429 | .668 | 1.000 |
|  |  | Head+Neck-Foreleg | 2.455 | .477 | 5.149 | .000 | .000 |
|  |  | Abdomen-Thorax | .341 | .477 | .715 | .475 | 1.000 |
|  |  | Abdomen-Foreleg | 2.591 | .477 | 5.435 | .000 | .000 |
|  |  | Thorax-Foreleg | 2.250 | .477 | 4.720 | .000 | .000 |
|  |  | Abdomen-Head+Neck | .136 | .477 | .286 | .775 | 1.000 |
|  | 3-5.08 | Hindleg-Head+Neck | -.577 | .439 | -1.316 | .188 | 1.000 |
|  |  | Hindleg-Abdomen | .000 | .439 | .000 | 1.000 | 1.000 |
|  |  | Hindleg-Thorax | -.885 | .439 | -2.017 | .044 | .437 |
|  |  | Hindleg-Foreleg | 2.865 | .439 | 6.534 | .000 | .000 |
|  |  | Head+Neck-Thorax | -.308 | .439 | -.702 | .483 | 1.000 |
|  |  | Head+Neck-Foreleg | 2.288 | .439 | 5.218 | .000 | .000 |
|  |  | Abdomen-Thorax | .885 | .439 | 2.017 | .044 | .437 |
|  |  | Abdomen-Foreleg | 2.865 | .439 | 6.534 | .000 | .000 |
|  |  | Thorax-Foreleg | 1.981 | .439 | 4.517 | .000 | .000 |
|  |  | Abdomen-Head+Neck | .577 | .439 | 1.316 | .188 | 1.000 |
|  | 17-19.08 | Hindleg-Head+Neck | -.661 | .423 | -1.564 | .118 | 1.000 |
|  |  | Hindleg-Abdomen | .000 | .423 | .000 | 1.000 | 1.000 |
|  |  | Hindleg-Thorax | -.696 | .423 | -1.648 | .099 | .993 |
|  |  | Hindleg-Foreleg | 2.839 | .423 | 6.719 | .000 | .000 |
|  |  | Head+Neck-Thorax | -.036 | .423 | -.085 | .933 | 1.000 |
|  |  | Head+Neck-Foreleg | 2.179 | .423 | 5.155 | .000 | .000 |
|  |  | Abdomen-Thorax | .696 | .423 | 1.648 | .099 | .993 |
|  |  | Abdomen-Foreleg | 2.839 | .423 | 6.719 | .000 | .000 |
|  |  | Thorax-Foreleg | 2.143 | .423 | 5.071 | .000 | .000 |
|  |  | Abdomen-Head+Neck | .661 | .423 | 1.564 | .118 | 1.000 |
|  | 31.08-2.09 | Hindleg-Head+Neck | -.547 | .395 | -1.383 | .167 | 1.000 |
|  |  | Hindleg-Abdomen | -.266 | .395 | -.672 | .502 | 1.000 |
|  |  | Hindleg-Thorax | -1.172 | .395 | -2.965 | .003 | .030 |
|  |  | Hindleg-Foreleg | 2.781 | .395 | 7.036 | .000 | .000 |
|  |  | Head+Neck-Thorax | -.625 | .395 | -1.581 | .114 | 1.000 |
|  |  | Head+Neck-Foreleg | 2.234 | .395 | 5.653 | .000 | .000 |
|  |  | Abdomen-Thorax | .906 | .395 | 2.293 | .022 | .219 |
|  |  | Abdomen-Foreleg | 2.516 | .395 | 6.364 | .000 | .000 |
|  |  | Thorax-Foreleg | 1.609 | .395 | 4.071 | .000 | .000 |
|  |  | Abdomen-Head+Neck | .281 | .395 | .712 | .477 | 1.000 |
|  | 14-16.09 | Hindleg-Head+Neck | -.433 | .408 | -1.061 | .288 | 1.000 |
|  |  | Hindleg-Abdomen | -.967 | .408 | -2.368 | .018 | .179 |
|  |  | Hindleg-Thorax | -1.667 | .408 | -4.082 | .000 | .000 |
|  |  | Hindleg-Foreleg | 3.267 | .408 | 8.002 | .000 | .000 |
|  |  | Head+Neck-Abdomen | -.533 | .408 | -1.306 | .191 | 1.000 |
|  |  | Head+Neck-Thorax | -1.233 | .408 | -3.021 | .003 | .025 |
|  |  | Head+Neck-Foreleg | 2.833 | .408 | 6.940 | .000 | .000 |
|  |  | Abdomen-Thorax | .700 | .408 | 1.715 | .086 | .864 |
|  |  | Abdomen-Foreleg | 2.300 | .408 | 5.634 | .000 | .000 |
|  |  | Thorax-Foreleg | 1.600 | .408 | 3.919 | .000 | .001 |
|  | Total | Hindleg-Head+Neck | -.985 | .383 | -2.569 | .010 | .102 |
|  |  | Hindleg-Abdomen | -1.309 | .383 | -3.413 | .001 | .006 |
|  |  | Hindleg-Thorax | -2.000 | .383 | -5.215 | .000 | .000 |
|  |  | Hindleg-Foreleg | 3.574 | .383 | 9.319 | .000 | .000 |
|  |  | Head+Neck-Abdomen | -.324 | .383 | -.844 | .399 | 1.000 |
|  |  | Head+Neck-Thorax | -1.015 | .383 | -2.646 | .008 | .081 |
|  |  | Head+Neck-Foreleg | 2.588 | .383 | 6.749 | .000 | .000 |
|  |  | Abdomen-Thorax | .691 | .383 | 1.802 | .071 | .715 |
|  |  | Abdomen-Foreleg | 2.265 | .383 | 5.906 | .000 | .000 |
|  |  | Thorax-Foreleg | 1.574 | .383 | 4.103 | .000 | .000 |
| Each row tests the null hypothesis that the Sample 1 and Sample 2 distributions are the same.  Asymptotic significances (2-sided tests) are displayed. The significance level is .050. | | | | | | | |
| a. Significance values have been adjusted by the Bonferroni correction for multiple tests. | | | | | | | |

## Mean intensity – foreleg regions – significant

| **Hypothesis Test Summary** | | | | | | |
| --- | --- | --- | --- | --- | --- | --- |
| Positive | Date | | Null Hypothesis | Test | Sig.^a,b^ | Decision |
| 1 | 28-30.09 | 1 | The distributions of As+Ad, Asc+Asd and Ase+Ade are the same. | Related-Samples Friedman's Two-Way Analysis of Variance by Ranks | .000 | Reject the null hypothesis. |
|  | 12-14.10 | 1 | The distributions of As+Ad, Asc+Asd and Ase+Ade are the same. | Related-Samples Friedman's Two-Way Analysis of Variance by Ranks | .002 | Reject the null hypothesis. |
|  | 26-28.10 | 1 | The distributions of As+Ad, Asc+Asd and Ase+Ade are the same. | Related-Samples Friedman's Two-Way Analysis of Variance by Ranks | 1.000 | Retain the null hypothesis. |
|  | 22-24.06 | 1 | The distributions of As+Ad, Asc+Asd and Ase+Ade are the same. | Related-Samples Friedman's Two-Way Analysis of Variance by Ranks | .441 | Retain the null hypothesis. |
|  | 6-8.07 | 1 | The distributions of As+Ad, Asc+Asd and Ase+Ade are the same. | Related-Samples Friedman's Two-Way Analysis of Variance by Ranks | .000 | Reject the null hypothesis. |
|  | 20-22.07 | 1 | The distributions of As+Ad, Asc+Asd and Ase+Ade are the same. | Related-Samples Friedman's Two-Way Analysis of Variance by Ranks | .000 | Reject the null hypothesis. |
|  | 3-5.08 | 1 | The distributions of As+Ad, Asc+Asd and Ase+Ade are the same. | Related-Samples Friedman's Two-Way Analysis of Variance by Ranks | .000 | Reject the null hypothesis. |
|  | 17-19.08 | 1 | The distributions of As+Ad, Asc+Asd and Ase+Ade are the same. | Related-Samples Friedman's Two-Way Analysis of Variance by Ranks | .000 | Reject the null hypothesis. |
|  | 31.08-2.09 | 1 | The distributions of As+Ad, Asc+Asd and Ase+Ade are the same. | Related-Samples Friedman's Two-Way Analysis of Variance by Ranks | .000 | Reject the null hypothesis. |
|  | 14-16.09 | 1 | The distributions of As+Ad, Asc+Asd and Ase+Ade are the same. | Related-Samples Friedman's Two-Way Analysis of Variance by Ranks | .000 | Reject the null hypothesis. |
|  | Total | 1 | The distributions of As+Ad, Asc+Asd and Ase+Ade are the same. | Related-Samples Friedman's Two-Way Analysis of Variance by Ranks | .000 | Reject the null hypothesis. |
| a. The significance level is .050. | | | | | | |
| b. Asymptotic significance is displayed. | | | | | | |

| **Related-Samples Friedman's Two-Way Analysis of Variance by Ranks Summary** | | | |
| --- | --- | --- | --- |
| 1 | 28-30.09 | Total N | 31 |
|  |  | Test Statistic | 35.109 |
|  |  | Degree Of Freedom | 2 |
|  |  | Asymptotic Sig.(2-sided test) | .000 |
|  | 12-14.10 | Total N | 15 |
|  |  | Test Statistic | 12.049 |
|  |  | Degree Of Freedom | 2 |
|  |  | Asymptotic Sig.(2-sided test) | .002 |
|  | 26-28.10 | Total N | 5 |
|  |  | Test Statistic | .000^a^ |
|  |  | Degree Of Freedom | 2 |
|  |  | Asymptotic Sig.(2-sided test) | 1.000 |
|  | 22-24.06 | Total N | 3 |
|  |  | Test Statistic | 1.636^a^ |
|  |  | Degree Of Freedom | 2 |
|  |  | Asymptotic Sig.(2-sided test) | .441 |
|  | 6-8.07 | Total N | 12 |
|  |  | Test Statistic | 15.943 |
|  |  | Degree Of Freedom | 2 |
|  |  | Asymptotic Sig.(2-sided test) | .000 |
|  | 20-22.07 | Total N | 22 |
|  |  | Test Statistic | 21.432 |
|  |  | Degree Of Freedom | 2 |
|  |  | Asymptotic Sig.(2-sided test) | .000 |
|  | 3-5.08 | Total N | 26 |
|  |  | Test Statistic | 39.915 |
|  |  | Degree Of Freedom | 2 |
|  |  | Asymptotic Sig.(2-sided test) | .000 |
|  | 17-19.08 | Total N | 28 |
|  |  | Test Statistic | 37.962 |
|  |  | Degree Of Freedom | 2 |
|  |  | Asymptotic Sig.(2-sided test) | .000 |
|  | 31.08-2.09 | Total N | 32 |
|  |  | Test Statistic | 37.000 |
|  |  | Degree Of Freedom | 2 |
|  |  | Asymptotic Sig.(2-sided test) | .000 |
|  | 14-16.09 | Total N | 30 |
|  |  | Test Statistic | 37.067 |
|  |  | Degree Of Freedom | 2 |
|  |  | Asymptotic Sig.(2-sided test) | .000 |
|  | Total | Total N | 34 |
|  |  | Test Statistic | 44.176 |
|  |  | Degree Of Freedom | 2 |
|  |  | Asymptotic Sig.(2-sided test) | .000 |
| a. Multiple comparisons are not performed because the overall test retained the null hypothesis of no differences. | | | |

| **Pairwise Comparisons** | | | | | | | |
| --- | --- | --- | --- | --- | --- | --- | --- |
| Positive | Date | Sample 1-Sample 2 | Test Statistic | Std. Error | Std. Test Statistic | Sig. | Adj. Sig.^a^ |
| 1 | 28-30.09 | Asc+Asd-Ase+Ade | -.613 | .254 | -2.413 | .016 | .047 |
|  |  | Asc+Asd-As+Ad | 1.468 | .254 | 5.779 | .000 | .000 |
|  |  | Ase+Ade-As+Ad | .855 | .254 | 3.366 | .001 | .002 |
|  | 12-14.10 | Asc+Asd-Ase+Ade | -.667 | .365 | -1.826 | .068 | .204 |
|  |  | Asc+Asd-As+Ad | 1.033 | .365 | 2.830 | .005 | .014 |
|  |  | Ase+Ade-As+Ad | .367 | .365 | 1.004 | .315 | .946 |
|  | 6-8.07 | Asc+Asd-Ase+Ade | -.500 | .408 | -1.225 | .221 | .662 |
|  |  | Asc+Asd-As+Ad | 1.375 | .408 | 3.368 | .001 | .002 |
|  |  | Ase+Ade-As+Ad | .875 | .408 | 2.143 | .032 | .096 |
|  | 20-22.07 | Asc+Asd-Ase+Ade | -.227 | .302 | -.754 | .451 | 1.000 |
|  |  | Asc+Asd-As+Ad | 1.205 | .302 | 3.995 | .000 | .000 |
|  |  | Ase+Ade-As+Ad | .977 | .302 | 3.241 | .001 | .004 |
|  | 3-5.08 | Asc+Asd-Ase+Ade | -.038 | .277 | -.139 | .890 | 1.000 |
|  |  | Asc+Asd-As+Ad | 1.462 | .277 | 5.270 | .000 | .000 |
|  |  | Ase+Ade-As+Ad | 1.423 | .277 | 5.131 | .000 | .000 |
|  | 17-19.08 | Asc+Asd-As+Ad | 1.250 | .267 | 4.677 | .000 | .000 |
|  |  | Ase+Ade-As+Ad | 1.482 | .267 | 5.546 | .000 | .000 |
|  |  | Ase+Ade-Asc+Asd | .232 | .267 | .869 | .385 | 1.000 |
|  | 31.08-2.09 | Asc+Asd-Ase+Ade | -.047 | .250 | -.187 | .851 | 1.000 |
|  |  | Asc+Asd-As+Ad | 1.266 | .250 | 5.063 | .000 | .000 |
|  |  | Ase+Ade-As+Ad | 1.219 | .250 | 4.875 | .000 | .000 |
|  | 14-16.09 | Asc+Asd-Ase+Ade | -.467 | .258 | -1.807 | .071 | .212 |
|  |  | Asc+Asd-As+Ad | 1.533 | .258 | 5.939 | .000 | .000 |
|  |  | Ase+Ade-As+Ad | 1.067 | .258 | 4.131 | .000 | .000 |
|  | Total | Asc+Asd-Ase+Ade | -.324 | .243 | -1.334 | .182 | .547 |
|  |  | Asc+Asd-As+Ad | 1.529 | .243 | 6.306 | .000 | .000 |
|  |  | Ase+Ade-As+Ad | 1.206 | .243 | 4.972 | .000 | .000 |
| Each row tests the null hypothesis that the Sample 1 and Sample 2 distributions are the same.  Asymptotic significances (2-sided tests) are displayed. The significance level is .050. | | | | | | | |
| a. Significance values have been adjusted by the Bonferroni correction for multiple tests. | | | | | | | |
